# Comprehensive investigation of SARS-CoV-2 intestinal pathogenesis in *Drosophila*

**DOI:** 10.1016/j.isci.2026.115621

**Published:** 2026-04-27

**Authors:** Layla El Kamali, Peter Nagy, Justine Girard, Nicolas Buchon, Patrick Mavingui, Chaker El-Kalamouni, Dani Osman

**Affiliations:** 1 University of La Réunion, INSERM U1187, CNRS UMR 9192, IRD UMR 249, Unité Mixte Processus Infectieux en Milieu Insulaire Tropical (UMR PIMIT), Plateforme Technologique CYROI, 97490 Sainte Clotilde, France; 2 Lebanese University, Faculty of Sciences III and Azm Center for Research in Biotechnology and its Applications (LBA3B, EDST), Tripoli, Lebanon; 3 Cornell Institute of Host-Microbe Interactions and Disease, Department of Entomology, Cornell University, 129 Garden Avenue, Ithaca, NY 14853, USA

**Keywords:** virology, microbiology

## Abstract

Gastrointestinal (GI) manifestations have been increasingly reported in patients with COVID-19. Here, we use the *Drosophila melanogaster* midgut model to investigate SARS-CoV-2-induced GI pathogenesis. The fly midgut exhibits susceptibility to orally administered virus, resulting in disrupted epithelial architecture, reduced organ size, and altered visceral muscle dynamics. These effects are accompanied by sustained proliferation of intestinal stem cells alongside decreased replenishment and viability of differentiated cells. Transcriptomic profiling reveals biphasic perturbations in midgut gene expression, particularly in pathways related to lipid metabolism. Intriguingly, SARS-CoV-2 elicits a dichotomous effect on lipid homeostasis, with lipid droplet accumulation in the posterior midgut and depletion in anterior segments. Treatment with Plitidepsin, a COVID-19 drug candidate, mitigates most SARS-CoV-2 pathogenic features in both the *Drosophila* midgut and human pulmonary cells, while modulating basal lipid droplet homeostasis in uninfected conditions. These findings establish the *Drosophila* midgut as a potent model for studying SARS-CoV-2 GI pathogenesis and evaluating antiviral compounds.

## Highlights

• SARS-CoV-2 ingestion rapidly disrupts *Drosophila* midgut structure and function
• Virus induces intestinal stem cell proliferation and impaired differentiation
• Midgut transcriptomes reveal biphasic remodeling with strong lipid metabolic shifts
• Plitidepsin mitigates SARS-CoV-2-induced gut pathophysiology

## Graphical abstract

**Figure:**
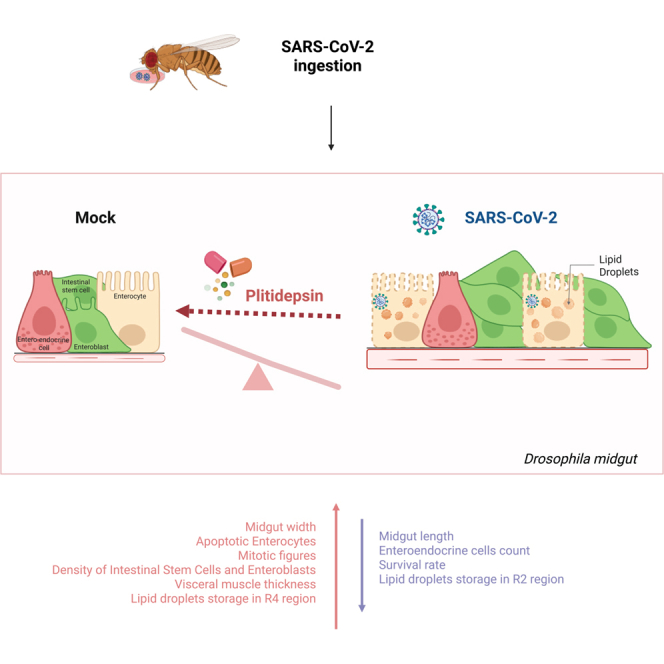

## Introduction

Coronaviruses (CoVs), a group of enveloped positive-sense single-stranded RNA viruses, predominantly affect birds and mammals, causing a spectrum of diseases ranging from mild to fatal.^1^^,^^2^ These viruses target the epithelial cells of the gastrointestinal (GI) and airway tracts predominantly, causing gastroenteritis and respiratory illnesses across various animal species, including wild, domestic, and farmed populations.^3^ Certain CoVs have breached the interspecies barrier, leading to zoonotic diseases, with the severe acute respiratory syndrome coronavirus 2 (SARS-CoV-2) being a prime example.^4^ This virus, belonging to the β-coronavirus genus, has been identified as the causative agent of the global pandemic known as coronavirus disease 2019 (COVID-19).

While respiratory complications are the primary clinical concern in SARS-CoV-2 infections, emerging evidence indicates the virus’s potential to disrupt multiple organ systems, particularly the digestive system.^5^ Cohort studies have documented a significant prevalence of GI symptoms, such as diarrhea, vomiting, loss of appetite, and abdominal discomfort, in patients with COVID-19, with occurrences ranging from 12 to 61% (reviewed in.^5^). Intriguingly, these symptoms can manifest before or even in the absence of respiratory signs,^6^ suggesting a direct viral invasion of the GI tract as an alternative infection route. This is further supported by the expression of the angiotensin-converting enzyme 2 (ACE2) receptor, which acts as the initial cell-virus contact point, and the transmembrane serine protease 2 (TMPRSS2), which primes the viral spike protein for entry, in GI epithelial tissues.^7^^,^^8^^,^^9^ This hypothesis is bolstered by the prolonged detection of viral antigens and RNA in GI biopsies and stool samples of infected individuals, pointing to a potential fecal-oral transmission route.^10^^,^^11^^,^^12^

Animal models for COVID-19, involving non-human primates and other vertebrates, have reinforced the evidence of the virus’ ability to infect and replicate in the digestive system, leading to extended periods of viral shedding in feces [reviewed in.^13^]. *In vitro* and *ex vivo* investigations, exploiting human intestinal epithelial cells, organoids, and human gut-on-chip models, have provided insights into SARS-CoV-2-induced GI pathogenesis.^14^^,^^15^^,^^16^^,^^17^ These studies highlight critical aspects of the infection outcomes, such as intestinal cell damage, the elicitation of local immune and inflammatory responses, with pronounced alterations in gene expression profiles, emphasizing the need for comprehensive research to elucidate the dynamic processes responsible for the GI complications associated with SARS-CoV-2 infection.

In this framework, the fruit fly *Drosophila melanogaster* emerges as an invaluable model for exploring the pathogenesis of SARS-CoV-2. This is mainly attributed to the substantial genetic overlap between the fruit fly and humans, with around 90% of human proteins known to interact with the virus being conserved within the fly genome.^18^ Recent advancements have led to the development of a *Drosophila* resource that enables tissue-specific co-expression of SARS-CoV-2 proteins alongside their corresponding human or fly binding partners, facilitating comprehensive *in vivo* analyses of viral-host interactions.^19^ Earlier work demonstrated that transgenic flies expressing SARS-CoV-2 proteins such as Orf6, Nsp6, and Orf7, exhibit reduced survival rates and display significant anomalies in tracheal development and muscle architecture.^18^ Particularity, ectopic Nsp6 expression causes increased glycolysis coupled to detrimental structural and functional alterations in the fly cardiac system, akin to some clinical manifestations observed in patients with COVID-19.^20^ Furthermore, the application of Selinexor, a nuclear transport inhibitor, mitigates the adverse effects of viral protein Orf6, highlighting *Drosophila's* potential for antiviral drug screening.^18^

The current study leverages the *Drosophila melanogaster* midgut model to investigate SARS-CoV-2-induced intestinal pathophysiology. The *Drosophila* midgut shares structural and functional similarities with the human small intestine, serving as the primary site for food digestion and nutrient absorption.^21^ Additionally, the intestinal epithelium acts as a barrier, safeguarding the organism from external invasive pathogens.^22^^,^^23^^,^^24^ Our approach involved orally exposing flies to a clinical isolate of the original pandemic variant and characterizing its impact on the midgut homeostasis and physiology during the early stages of infection. Furthermore, our study demonstrated the effectiveness of this SARS-CoV-2 midgut model in evaluating the activity of promising anti-SARS-CoV-2 candidates such as Plitidepsin,^25^ and in establishing parallels with human pulmonary cells.

## Results

### SARS-CoV-2 administration by oral route compromises *Drosophila* survival

To assess the susceptibility of *Drosophila melanogaster* to SARS-CoV-2 via the oral route, wild-type mated females (*w*^*1118*^) aged 2–3 days, initially reared at 18°C, were shifted to 29°C for two additional days before virus ingestion and kept at this temperature during the experiments. This temperature was chosen to facilitate subsequent genetic manipulations and ensure the viability of the flies, while potentially maintaining the stability of the virus.^26^ On the day of exposure (D0), flies were starved for 2 h to synchronize feeding behavior (Figure S1A). Flies that received the SARS-CoV-2 inoculum are denoted as ingested, with time points given as hours/days postingestion (hpi/dpi); mock denotes vehicle-treated controls.

**Figure 1:**
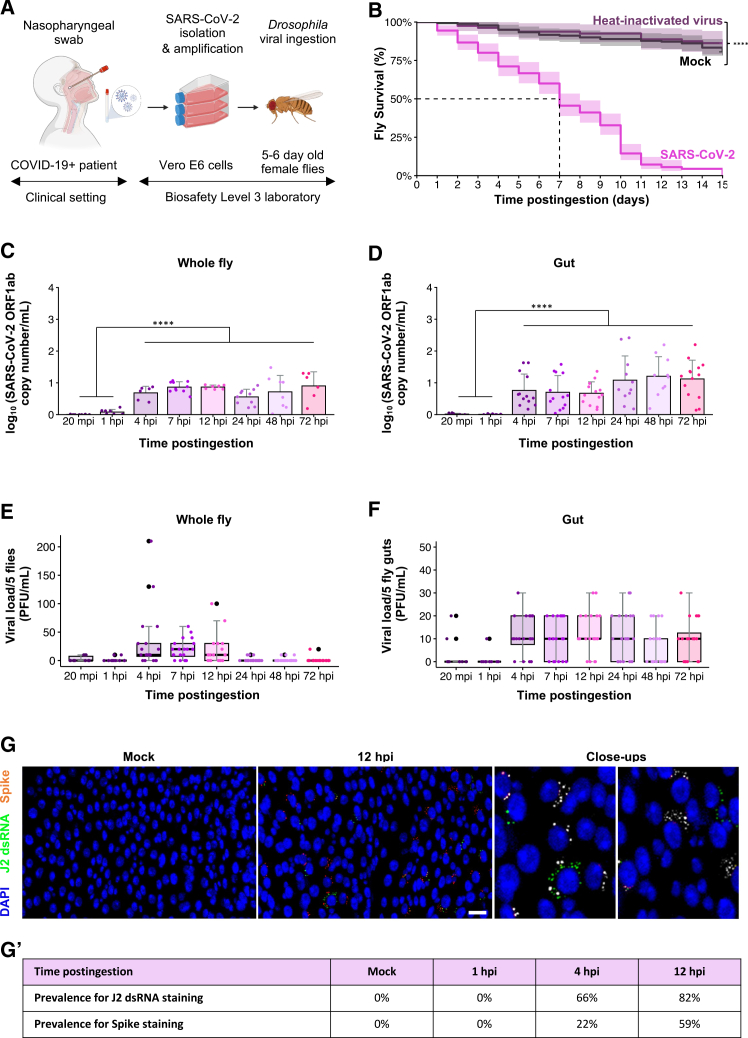
*Drosophila* susceptibility and permissiveness to SARS-CoV-2 ingestion (A) Study design: 5–6-day-old female flies were orally administered a solution of SARS-CoV-2, obtained from a nasopharyngeal swab of a COVID-19 patient. The clinical strain was isolated and amplified on Vero E6 cells. Created with BioRender. (B) Kaplan-Meier survival curves compare flies (*w*^*1118*^ genetic background) that ingested SARS-CoV-2, mock flies that ingested Vero E6 cell supernatants, and those exposed to heat-inactivated SARS-CoV-2. Experiments represent three biological replicates, with 180 flies per condition in each replicate (total *n* = 540 flies per condition). Shaded areas represent the 95% confidence intervals (CIs). Statistical significance was measured using a global Log rank test (*p* < 0.01). (C) Longitudinal detection of SARS-CoV-2 genomic RNA using the open-reading frame 1 ab (ORF1ab) RT-qPCR assay. Samples consisted of pools of 5 ground whole flies or guts. (D). Mock-treated flies tested negative at all time points. Three independent experiments were conducted with 4 samples each (*n* = 12 samples/condition/timepoint). Histograms show means with error bars indicate standard deviation. *p*-values correspond to the significant difference between different times of postingestion and were calculated using unpaired t-tests, corrected according to the Bonferroni method. Significance levels are: ∗∗∗∗*p* < 0.0001. (E) Longitudinal quantification of infectious viral loads using plaque-forming assay (PFU). Samples consisted of pools of 5 ground whole flies or guts. (F). Mock-treated flies tested negative at all time points. Three independent experiments were performed with 6 samples each (*n* = 18 samples/condition/timepoint). Each dot represents values from one sample, larger black dots mark outliers. (G) Representative images of a midgut confocal section dissected at 12 hpi, with its respective control. Midguts were stained with anti-SARS-CoV-2 spike antibody (red), anti-dsRNA J2 antibody (green), and DAPI (blue). Midguts from mock-treated flies revealed no signal over time. Three independent experiments were performed with 6 samples each (*n* = 18 samples/condition/timepoint). Scale bars represent 50 μm. In the close-up views, Spike staining is shown in white to enhance contrast. (G′) Prevalence of cells positive for J2 dsRNA and Spike staining quantified at 1, 4, and 12 hpi. Data represent the mean of three independent experiments.

The clinical isolate used in this study corresponds to the 2020 European D614G SARS-CoV-2 variant, derived from a nasopharyngeal swab and passaged once on Vero E6 cells (Figure 1A). The full-length genome sequence of this isolate is publicly available on GISAID under accession ID: RUN-PIMIT8.^27^ Infectivity of the viral stock was confirmed by immunostaining with an anti-SARS-CoV-2 spike-specific antibody (Figure S1B), and by monitoring cytopathic effects in Vero E6 cells (Figure S1C). For oral infection, flies were exposed to a dose of 0.45x10^6^ plaque-forming units (PFUs) (Figure S1D); mock controls received cell culture supernatant from uninfected cells.

Survival was markedly reduced in flies orally challenged with SARS-CoV-2 relative to mock-treated controls (log rank test, *p*-value<0.001, Figure 1B). Mortality increased over time, reaching 50% by day 7 postingestion and ∼90% by day 10. To rule out effects of nonviral components in the inoculum, we included a heat-inactivated virus control (60°C, 30 min), which eliminates infectivity while preserving protein/medium composition. Plaque assays confirmed no residual infectivity after heat treatment. Flies exposed to the heat-inactivated preparation showed survival comparable to mock controls (log rank test, *p*-value>0.5, Figure 1B). To capture early host responses before survival diverged, subsequent analyses were mainly focused on the first 72 hpi, providing a window on the initiation and early progression of disease.

To monitor viral presence, RT-qPCR was performed on total RNA from whole flies and dissected midguts. SARS-CoV-2 genomic RNA (ORF1ab and the Nucleocapsid protein N) genes were detectable as early as 4 hpi and persisted through 72 hpi (Figures 1C, 1D, S1E, and S1F). Viral RNA levels showed no significant variation across the different time points (85% ± 10% of the samples testing positive; one-way ANOVA, *p*-value>0.5). Mean cycle-threshold (Ct) values were 28 (ORF1ab) and 26 (N). At very early time points (20 min and 1 hpi), when the signal likely reflects residual inoculum, viral RNA was below the detection threshold (Figures 1C, 1D, S1E and S1F).

To assess infectious virus, PFU assays were conducted on homogenates from whole flies and isolated midguts. Despite technical constraints associated with applying fly homogenates to cell cultures, infectious particles were recovered from whole flies at 4–12 hpi, with an estimated titer of ∼15 PFU/mL (Figure 1E). In midgut samples, mean titers were ∼10 PFU/mL across tested time points, with ∼75% of samples yielding detectable infectious virus (Figure 1F). At the earliest time points postexposure, infectious virus was below the limit of detection in both whole-fly and gut homogenates (Figures 1E and 1F), indicating that later PFU reflect *de novo* production rather than residual inoculum.

Immunofluorescence staining for the Spike protein showed widespread distribution of viral antigen along the digestive tract, with a gut-wide prevalence of 72.2% ± 5.56% across timepoints (Figure 1G). To probe replication, we stained with the J2 antibody, which recognizes double-stranded RNA intermediates. Cells co-labeled for Spike and J2 were observed (not all signals colocalized), with increasing prevalence over time (Figure 1G’), consistent with viral entry and replication in gut epithelial cells.

To assess horizontal transmission, *W*^*−*^ (white-eyed) flies were orally exposed to SARS-CoV-2 for 12 h and then co-housed with unexposed *W*^*+*^ (red-eyed) flies in the same vials (Figure S1G), enabling visual discrimination of the two populations with shared food surfaces and close contact. Survival was monitored in both groups. Under our conditions, *W*^*+*^ flies maintained normal viability throughout the assay (Figure S1G’), indicating no detectable horizontal transmission. Collectively, our data show that *Drosophila* is susceptible to oral SARS-CoV-2 exposure, with early mortality, persistent viral RNA, limited but detectable infectivity, and epithelial tropism. Although productive replication appears low, the consistent presence of viral RNA and virulence-associated mortality supports *Drosophila* as a tractable *in vivo* model for probing early-stage interactions between SARS-CoV-2 virulence factors and the GI epithelium.

### SARS-CoV-2 ingestion causes structural and physiological perturbations in the *Drosophila* intestine

**Figure 2:**
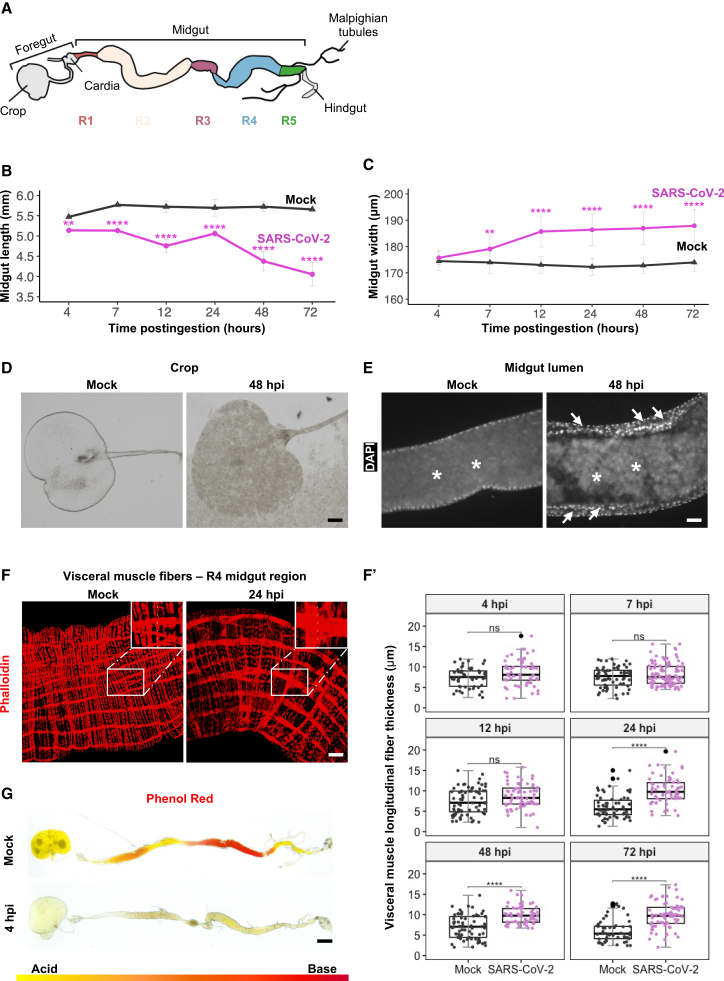
Effects of SARS-CoV-2 ingestion on midgut structure and pH in *Drosophila* (A) Schematic representation of the adult *Drosophila* digestive tract. The midgut is subdivided into five functionally and morphologically distinct regions (R1-R5), each represented in a different color. (B and C) Measurements of gut length and width at different timepoints postingestion (*n* = 18 midguts/condition/timepoint). Error bars indicate standard deviation. *p*-values correspond to the significant difference between conditions at the same time postingestion and were calculated using simple t-tests, corrected according to the Bonferroni method. Significance levels are: ∗∗*p* < 0.01 and ∗∗∗∗*p* < 0.0001. Absence of an asterisk indicates no significant difference (*p* ≥ 0.05). (D) Representative images of crops from infected and mock flies at 48 hpi. Observations were performed across three independent experiments, with six crops analyzed per experiment (*n* = 18 crops per condition per time point). Scale bars represent 30 μm. (E) Images focus on midgut lumens (R4 region; *n* = 6, 3 replicates) of virus-exposed and mock flies. Guts were stained with DAPI at 48 hpi. Asterisks indicate the intestinal lumen, and arrows point to the thickened intestinal epithelium. Scale bars represent 30 μm. (F) Longitudinal visceral muscle fibers (R4 region) of infected and mock flies, stained with Phalloidin (red) at 24 hpi. Scale bars represent 50 μm. (F′) Quantitative measurements of muscle fiber thickness across the R4 region of dissected midguts at different times postingestion. Each dot represents measurements from a single longitudinal fiber. *p*-values are ns > 0.05, ∗∗∗∗ <0.0001 calculated by Mann–Whitney U test. (G) Full guts of wild type *w*^*1118*^ flies fed with either mock or viral solutions mixed with Phenol red dye at 4 hpi (*n* = 6 per condition, 3 independent replicates). Yellow indicates acidic regions (pH ≤ 6), orange is neutral (6<pH < 8), and red is alkaline regions (pH > 8). Scale bars represent 350 μm.

Following oral exposure to SARS-CoV-2, pronounced morphological changes became evident in the midgut (Figure 2). Measurements of the midgut length, taken from the cardia center to the midgut/hindgut junction (Figures 2A and 2B), revealed a significant and constant decrease in size compared with controls, which maintained a consistent length of approximately 5.65 ± 0.15 mm. Midgut shortening was detectable as early as 4 hpi and became more pronounced over time, reaching ∼70% of the initial length by later timepoints (Figure 2B). In parallel, midgut width increased significantly starting at 7 hpi (Figure 2C). Because width naturally varies along the midgut, measurements were standardized within region R4. These morphological alterations were coupled with intestinal obstructions, particularly evident at 48 hpi, where they were observed in 83% of the samples. Obstructions were characterized by the build-up of food materials in the hypertrophied crop (Figure 2D) and within the lumen of infected guts (Figure 2E), likely interfering with food processing and peristaltic function.

To further investigate gut motility, we visualized F-actin filaments in visceral muscles using phalloidin staining. Marked abnormalities in the muscular architecture were observed (Figure 2F), including a significant thickening of longitudinal syncytial muscle fibers throughout the midgut beginning at 24 hpi (Figure 2F’). This thickening, which persisted for at least 72 hpi (Figure 2F’), suggests potential visceral muscle spasms that may contribute to the observed midgut shortening. Alongside these structural modifications, SARS-CoV-2 ingestion led to a swift acidification of the entire digestive tract as early as 4 hpi (Figure 2G).

To evaluate intestinal transit, bromophenol blue (BPB) was added to both the mock and SARS-CoV-2 solutions, producing a visibly blue ingested medium. Fecal output was quantified by counting the number of blue excreta spots per vial. To ensure accuracy given the high defecation rate, flies were transferred to new vials at 1 hpi and 4 hpi, and measurements were recorded at 1, 4, and 12 hpi (Figure S2A). Control flies displayed a regular distribution of fecal spots, whereas SARS-CoV-2-exposed flies exhibited a marked reduction in fecal output, consistent with impaired intestinal transit or partial obstruction (Figure S2A’).

Other organs, namely the rectum and ovaries, were also affected by SARS-CoV-2 ingestion (Figures S2B and S2C). Approximately 90% of the examined recta were hypertrophied, with surface area increased by 200% ± 20% relative to mock-treated controls and a concomitant loss of clear papillar segmentation, most pronounced at 72 hpi (Figure S2B). The ovaries likewise showed structural disorganization and overall size reduction (Figure S2C). Whether these changes reflect direct viral replication or indirect systemic effects remains unresolved.

Together, these findings highlight the profound impact of SARS-CoV-2 enteric exposure on the *Drosophila* model, characterized by notable structural and physiological changes within the digestive tract and other organs. These observations elucidate the complex nature of SARS-CoV-2 pathogenesis in this experimental system.

### SARS-CoV-2 leads to disruptions in the cellular composition of the *Drosophila* midgut

**Figure 3:**
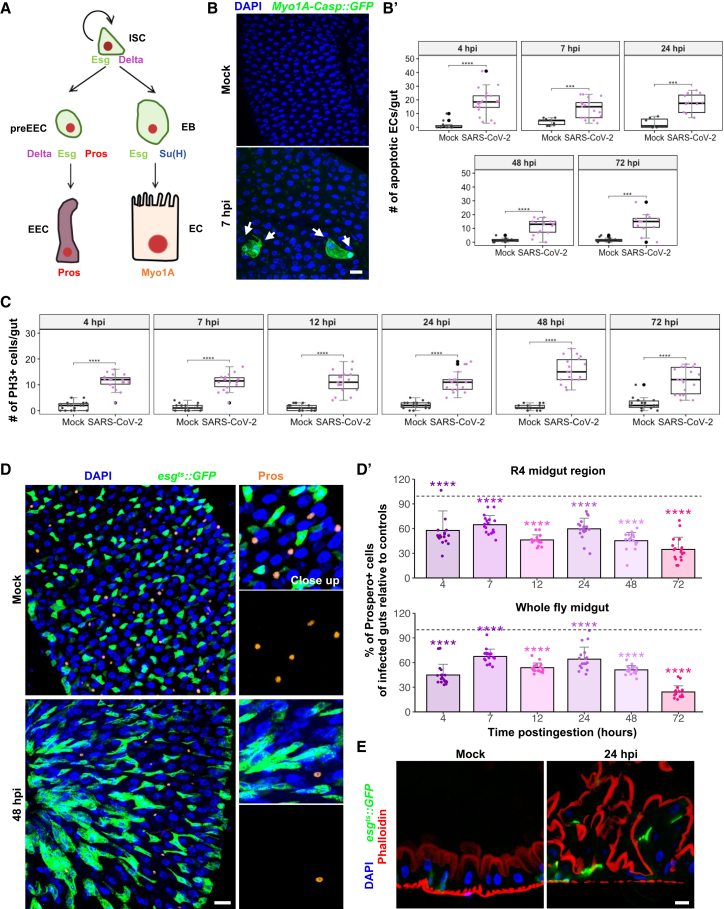
Disruptions in midgut cellular composition upon SARS-CoV-2 ingestion (A) Schematic representation of the *Drosophila* adult intestinal stem cell lineage with cell specific markers. (B) Representative images of the R4 midgut region at 7 hpi show Caspase 3 activity (GFP) driven by *Myo1A-GAL4* in ECs, compared to its respective mock. Arrows point to apoptotic ECs. Scale bars represent 50 μm. Quantification of apoptotic ECs per midgut at different times postingestion is shown in (B′). (C) Quantification of mitotic ISCs (PH3-positive cells) per midgut at different times postingestion. (D) Representative images of the R4 midgut region at 48 hpi and its respective mock, showing ISCs and EBs (*esg*^*ts*^*::GFP*, green), and preEE/EE cells (Pros, red). Scale bars represent 50 μm. Close-ups show merged and separate channels for Pros. (D′) Percentage of Prospero-positive cells in the R4 region and in whole infected guts relative to their controls. Dashed lines indicate the mock baseline. Histograms show means of the ratios, and error bars indicate standard deviation. (E) Confocal sections of *esg*-positive midguts express GFP in ISCs and EBs. F-actin in the brush border microvilli was stained using Phalloidin. Scale bars represent 30 μm. Data were collected from three independent replicates with 6 midguts each (*n* = 18 midguts/condition/timepoint). In boxplots, each dot represents a count from one gut; large black dots mark outliers. *p*-values were calculated using simple t-tests corrected using the Bonferroni method. Statistical significance is indicated as ∗∗∗*p* < 0.001 and ∗∗∗∗*p* < 0.0001. DAPI is blue.

Having shown that SARS-CoV-2 ingestion impacts the structural integrity of the *Drosophila* alimentary canal, our investigation extended to the virus's potential influence on the cellular makeup of the midgut epithelium, which appears thicker after viral exposure (arrows in Figure 2E). This layer is primarily a single sheet composed predominantly of fully differentiated enterocytes (ECs) and enteroendocrine cells (EECs), which arise from progenitor cells known as enteroblasts (EBs) and pre-enteroendocrine cells (preEECs), respectively^28^ (Figure 3A). To maintain tissue integrity and function under physiological conditions, multipotent intestinal stem cells (ISCs) sporadically divide, yielding another ISC and either an EB or a preEEC. These cells can be distinguished by their expression of specific transcription factors: ISCs, EBs, and preEECs express Escargot (Esg), EBs are marked by suppressor of hairless (Su(H)), and both preEECs and EECs express Prospero (Pros) (Figure 3A). In response to tissue damages, such as bacterial infection, this intestinal homeostatic program accelerates to effectively replenish lost cells.^29^ The following section aims to discern whether SARS-CoV-2 exposure disrupts this delicate cellular balance within the midgut epithelium.

SARS-CoV-2 is well-documented for its capacity to induce apoptosis in mammalian cell cultures. A recent investigation by Chu et al.^30^ demonstrated that the virus strategically exploits caspase 6 in the apoptotic pathway to boost its replication efficiency. To assess the impact of SARS-CoV-2 on apoptosis specifically in ECs, the predominant midgut cell type, we used the *Myo1A>Casp::GFP* transgenic fly line. This line enables the expression of the Caspase 3 sensor, Casp::GFP, under the control of the *Myo1A-GAL4* EC-specific driver, as previously described.^31^^,^^32^ In this system, GFP fluorescence is an indicator of Caspase 3 activation within ECs. Our analysis revealed an uptick in apoptotic ECs across the midgut regions of flies exposed to SARS-CoV-2 compared to controls (Figure 3B). This increase in apoptotic activity was detected as early as 4 hpi and persisted at all subsequent timepoints (Figure 3B’).

Damage-induced cell death in the midgut usually triggers the proliferation of ISC populations to maintain tissue integrity.^33^ To test this hypothesis, we stained the midguts with an anti-PH3 antibody, which specifically marks phosphorylated histone H3 in condensed chromatin found in mitotic cells (Figure S3A). In the midguts challenged with SARS-CoV-2, we spotted a 10-fold increase in mitotic indexes as early as 4 hpi, compared to their mock counterparts (Figure 3C). Interestingly, the proliferation rate remained consistently high up to 72 hpi. Concomitant with the elevated mitotic activity, there was a noticeable increase in the number of *esg*^*+*^ progenitor cells (*esg*^*+*^*: esg-GAL4>UAS-GFP, tubP-GAL80*^*ts*^) throughout the midgut in response to SARS-CoV-2 enteric challenge (Figure 3D). This augmentation was confirmed by quantifying the density of *esg*^*+*^ cells within a defined 20,000 μm^2^ area of the midgut R4 region, starting from 4 hpi to 72 hpi (Figure S3B). The high ISC activity in challenged midguts was maintained in surviving flies at late days postexposure (Figures S3C and S3C′). Further confirmation of these findings came from observing an increase in the number of ISC-*Delta+* cells (*tubP-GAL80*^*ts*^*; Delta-GAL4>UAS-GFP*) in SARS-CoV-2 midguts at 7 and 48 hpi compared to controls (Figures S3D and S3D′).

The GI tract functions as an endocrine organ, primarily through the activity of EECs that secrete hormones essential for various physiological processes, including peristalsis, digestion, nutrient absorption, feeding behavior, and metabolism regulation.^21^ Given this critical role, we investigated the impact of SARS-CoV-2 on EEC populations. Utilizing an anti-Prospero antibody to specifically mark preEE and EE cells, we found that SARS-CoV-2 precipitates a significant decrease in the number of EECs in the midgut (Figure 3D). Notably, the EEC count was reduced by approximately 50% as early as 4 hpi and persisted until 72 hpi when compared to control midguts (Figure 3D’).

Taken together, these results demonstrate that SARS-CoV-2 disrupts intestinal cellular homeostasis, marked by increased apoptosis in ECs, elevated proliferation of ISCs, and a pronounced reduction in EEC numbers. This disruption is further evidenced by the altered structural organization and unusual multilayering of the intestinal epithelium (Figure 3E and schematized in Figures S3E and S3E′), as revealed by Phalloidin staining that highlights the apical brush borders of ECs.

### SARS-CoV-2 impacts the replenishment of differentiated cells within the midgut epithelium

To thoroughly investigate the fate of ISCs after SARS-CoV-2 enteric challenge, we performed cell lineage tracing experiments using the repressible dual differential stability marker (ReDDM) genetic system.^34^ The ReDDM methodology allows simultaneous expression of proteins with distinct half-lives: a short-lived mCD8:GFP and a long-lived H2B::RFP, under the regulation of cell-specific drivers, including *delta-GAL4* (*Delta-ReDDM*), *Su(H)-GAL4* (*Su(H)-ReDDM*), and *esg-GAL4* (*esg-ReDDM*). This enabled us to monitor the dynamic cell behavior in the midgut over time, as illustrated in Figures S4A–S4C.

**Figure 4:**
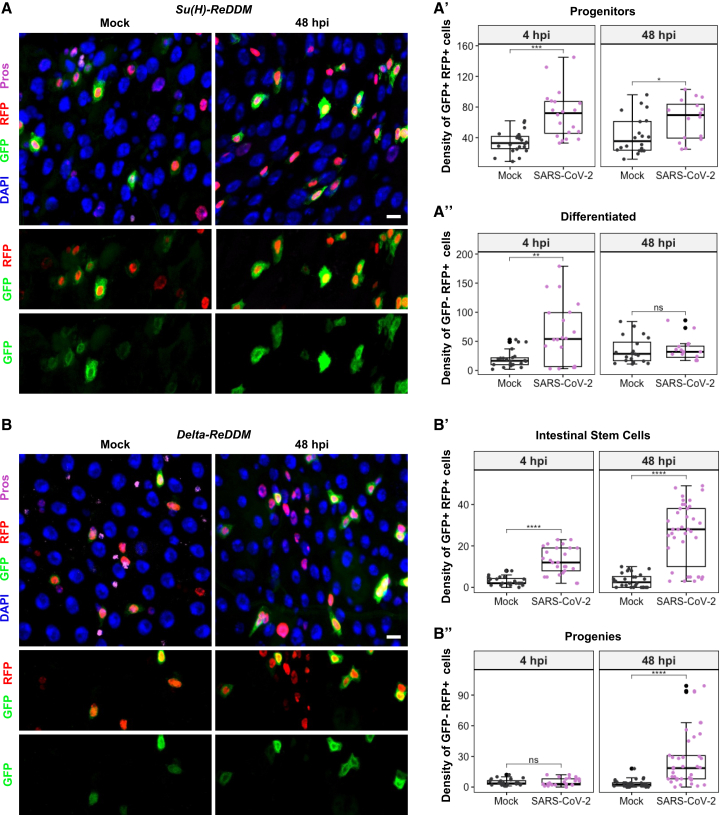
Impact of SARS-CoV-2 on midgut cellular regeneration and differentiation (A) Representative images of *Su(H)*-*ReDDM* at 48 hpi. (A′) Quantification of the density of progenitors (EBs = Su(H)+ = GFP+ RFP+ cells) and (A″) differentiated cells (ECs + EEs = RFP+ only cells). (B) Representative images of *Delta*-*ReDDM* at 48 hpi. (B′) Quantification of the density of intestinal stem cells (ISCs (+preEEs) = delta+ = GFP+ RFP+ cells), and (B″) their progenies (ECs + EEs + EBs = RFP+ only cells). DAPI is blue, and the scale bars represent 20 μm. Images were acquired at 48 hpi using confocal microscopy. Quantification was done in a specific surface area of 20,000 μm^2^ of R4 at 4- and 48 hpi. Data were collected from three independent replicates with 6 midguts each (*n* = 18 midguts/condition/timepoint). Each dot represents a count from one midgut, and large black dots mark outliers. *p*-values from the Mann–Whitney U-test are ns > 0.05, ∗ <0.05, ∗∗ <0.01, ∗∗∗ <0.001, and ∗∗∗∗ <0.0001.

Prior to viral challenge, 3-day-old mated females were conditioned at 29°C for 3 days, and kept until dissection, to activate cellular markers effectively. The *Su(H)-ReDDM* system highlighted a notable rise in the populations of *Su(H)*^*+*^ (EBs: GFP^+^, RFP^+^) at both 4 and 48 hpi, compared to controls (Figures 4A and 4A′). Although the count of newly differentiated ECs (GFP^−^, RFP^+^) was higher than control levels at 4 hpi, this did not translate into a sustained EC replenishment at 48 hpi (Figure 4A’’). When using the *Delta-ReDDM* genetic setup, *Delta*^*+*^ cells (ISCs and preEECs: GFP^+^, RFP^+^) displayed an increase in number at 4 hpi (Figures 4B and 4B′), aligning with earlier findings using the *Delta-GAL4*^*ts*^*>UAS-GFP* fly line (Figure S3D). However, their progeny, encompassing EBs, ECs, and EECs, did not show an increase in number at 4 hpi but demonstrated a significant rise in their numbers at 48 hpi (Figure 4B”), likely due to enhanced EB population as depicted with the *Su(H)-ReDDM* flies (Figures 4A and 4A′). Consistently, the *esg-ReDDM* system revealed a significant rise in the total count of *esg*^*+*^ cells (ISCs and EBs: GFP^+^, RFP^+^) after viral exposure compared to controls (Figures S4D and S4D′), yet without a simultaneous increase in the count of newly formed differentiated cells (GFP^−^, RFP^+^) (Figure S4D”).

Overall, our data reveal that SARS-CoV-2 compromises the regenerative processes within the *Drosophila* midgut epithelium, affecting the replenishment of ECs and EECs, despite the induction of continuous ISC proliferation and increased EB numbers. The observed impediment in the transition to fully differentiated cellular states postingestion underscores the necessity for further investigations to unravel the mechanisms driving these disruptions.

### Transcriptomic profiling reveals a biphasic molecular response in SARS-CoV-2-challenged midguts

**Figure 5:**
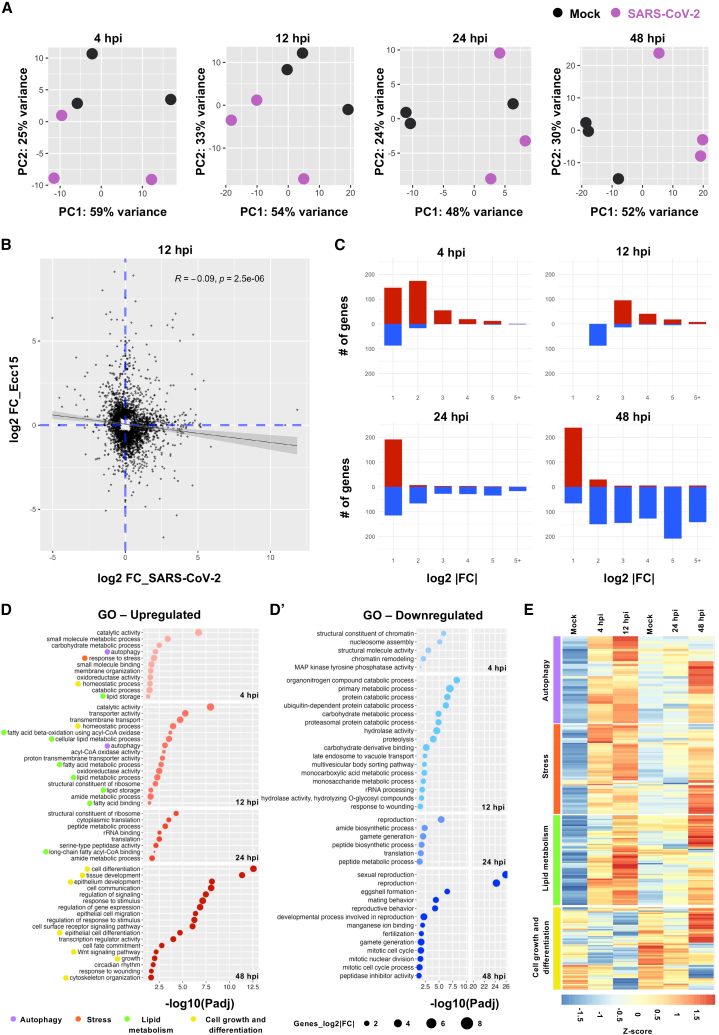
Biphasic molecular response to SARS-CoV-2 ingestion revealed by midgut transcriptomic profiling (A) Principal Component Analysis (PCA) of the transcriptomes of SARS-CoV-2 infected guts vs. mock at different times postingestion. Three independent replicates were considered per condition. (B) Scatterplot compares the log2 fold change of differentially expressed genes (DEGs) following *Erwina carotovora carotovora* (*Ecc15*) oral infection (y axis) and SARS-CoV-2 ingestion (x axis) at 12 hpi. (C) Transcriptomic variation in guts at different times postingestion, showing the number of upregulated (red) and downregulated (blue) genes categorized by the level of log2 fold change (log2FC). Only significantly DEGs (*p*-value<0.05) are shown in this graph. (D) Gene ontology (GO) enrichment bubble plot of upregulated and (D′) downregulated genes grouped by time postingestion. (E) Clustering heatmaps of gene expression across different times postingestion compared to their respective controls. Genes were clustered based on their GO categories.

To gain insights into the molecular dynamics of the response to SARS-CoV-2 enteric challenge in *Drosophila*, we conducted comprehensive transcriptomic profiling at 4, 12, 24, and 48 hpi against mock conditions (Figure S5A; Table S1). Principal component analysis (PCA) indicated global shifts of midgut transcriptomes at all timepoints postingestion (Figure 5A). The first principal component (PC1) accounted for 48–59% of the total variance, while the second principal component (PC2) accounted for 24–33% of the variance. This separation along the PC1 and PC2 axes emphasizes the substantial global differences in midgut transcriptomes in response to SARS-CoV-2 exposure over time.

Comparative analysis of midgut responses to pathogenic *Erwinia carotovora carotovora* (*Ecc15*) infection, which triggers the robust induction of immune, stress, and developmental pathways,^33^ revealed distinct global expression profiles. Fold change values for genes at multiple timepoints (4, 12, and 48 hpi) following SARS-CoV-2, and *Ecc15* ingestion showed no correlation (e.g., R^2^ = −0.09, *p* = 2.5 e+06 at 12 hpi), indicating a specific transcriptomic response to SARS-CoV-2 (Figures 5B and S5B; Table S2).

Gene Ontology (GO) analysis of differentially expressed genes revealed enrichment for metabolic and cellular process terms rather than canonical immune pathways (Figure S5C; Table S4), indicating a shift toward physiological and structural remodeling. Given this unconventional profile, we next examined temporal dynamics to track how these programs evolve over the course of ingestion. The early response to SARS-CoV-2 ingestion (4 and 12 hpi) was dominated by upregulation, most commonly 2- to 4-fold, with relatively few downregulated transcripts. As time progressed (24 and 48 hpi), the number of downregulated genes became more prominent, though some genes continued to show mild upregulation (less than 2-fold) (Figures 5C and S5D). A core set of genes consistently differentially expressed across timepoints emerged, with 307 genes upregulated and 60 downregulated at 4 and 12 hpi, versus 39 upregulated and 123 downregulated at 24 and 48 hpi (Figures S5E, S5E′ and S5F; Table S3). GO analysis revealed early upregulated genes (4 and 12 hpi) were predominantly associated with stress responses, autophagy, transmembrane transport, and lipid metabolism (Table S4; Figure 5D). At later stages (24 and 48 hpi), upregulated genes were mainly linked to translation (ribosomal constituents, rRNA binding), cell differentiation, epithelium development, growth, and cytoskeletal organization (Table S4; Figure 5D). Downregulated genes at early timepoints were predominantly involved in chromatin remodeling, proteolysis, ubiquitin-dependent proteasomal protein degradation, and late endosome/multivesicular body transport (Figure 5D’). At later stages, downregulated genes included those involved in translation (a different set from the upregulated genes at earlier timepoints), reproduction, and mitotic cell cycle progression (Figure 5D’). We further examined the temporal changes in expression levels of specific gene categories critical during viral infection. Genes related to autophagy, stress responses, and lipid metabolism showed early upregulation, peaking at 12 hpi, followed by a decrease at 24 hpi and only a subset peaking again at 48 hpi (Figure 5E). In contrast, genes involved in cell growth and differentiation showed a decrease at early time points, peaking at high expression levels by 48 hpi (Figure 5E). The rapid upregulation of the host genes likely reflects immediate gut defense mechanisms while the virus is being internalized and replicating. The subsequent downregulation of key cellular processes at later stages may reflect the virus’s strategy to hijack the host’s cellular machinery, ensuring viral propagation.

In summary, our findings reveal a biphasic molecular response to SARS-CoV-2 in the *Drosophila* midgut. Early responses are dominated by the upregulation of genes related to stress response, autophagy, and lipid metabolism, whereas later stages exhibit the downregulation of genes associated with translation and cell cycle progression. Understanding these molecular shifts provides crucial insights into the life cycle of SARS-CoV-2 and its interaction with intestinal host biology, which may inform the development of effective antiviral strategies.

### SARS-CoV-2 induces region-specific disruptions in midgut lipid droplet organelles

**Figure 6:**
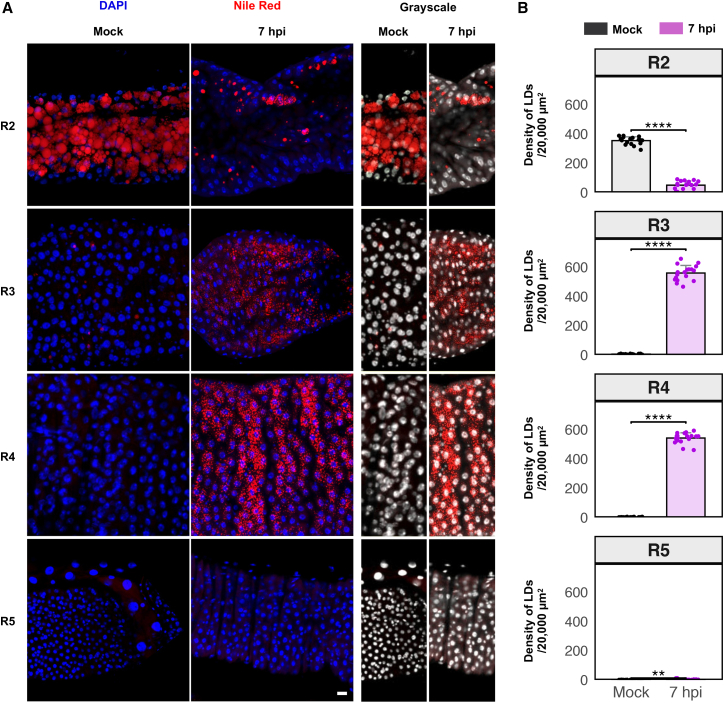
Region-specific disruptions in midgut lipid droplets homeostasis induced by SARS-CoV-2 (A) Representative images show the distribution of lipid droplets in R2 to R5 regions of SARS-CoV-2-infected midguts at 7 hpi, using Nile Red staining. Comparisons are made to their respective mock samples. Nuclei are counterstained with DAPI (blue; white in grayscale panels), and scale bars represent 20 μm. A total of 20 midguts were scored for this experiment. (B) Density of lipid droplet particles in a 20,000 μm^2^ surface area of the R2-R5 midgut regions at 7hpi compared to mock midguts (*n* = 6 per condition/region). Histograms show means, and error bars indicate standard deviation. *p*-values were defined using individual t-tests and corrected using the Bonferroni method for multiple comparisons (ns *p* > 0.05 and ∗∗∗*p* < 0.001).

Our transcriptomic analysis unveiled that SARS-CoV-2 promotes a marked transcriptional reprogramming of lipid metabolism genes in the early phase of enteric viral challenge (Figures 5D and 5E). To better visualize the relationships between differentially expressed genes and the lipid metabolic pathways they are involved in, we performed a KEGG pathway enrichment analysis on the subset of genes modulated at 12 hpi, illustrated using a chordDiagram (Figure S5F). This representation highlights the overlap among genes contributing to multiple lipid-related processes, such as glycerophospholipid metabolism, fatty acid degradation, and sphingolipid metabolism. Notably, several genes were simultaneously implicated in distinct metabolic routes, suggesting coordinated regulation and potential functional convergence during viral exposure. Building on this, we further explored the influence of SARS-CoV-2 on the homeostasis of intestinal lipid droplets (LDs), which are key cellular organelles involved in energy storage and lipid metabolism.^35^ This investigation also relies on prior findings showing that SARS-CoV-2 induces LD accumulation in pneumocytes of patients with COVID-19.^36^ Utilizing Nile Red and BODIPY stains, known for their efficacy in visualizing neutral lipids, we observed significant LD homeostasis disruptions within the *Drosophila* intestine at various postingestion intervals, notably at 7 and 12 hpi, as showcased in Figures 6A and S6A. These disruptions were heterogeneously distributed along the midgut, with pronounced, region-specific LD distribution variations (Figures 6A, 6B, and S6A). Consistent with a previous study,^37^ our data confirm the natural abundance and size heterogeneity of LDs in the R2 region under uninfected conditions. After oral SARS-CoV-2 exposure, however, there was a conspicuous depletion in both the number and size of LDs in this region, diverging markedly from the control. In the R3 and R4 regions, there was a noticeable accumulation of LDs postingestion. Intriguingly, even with the heightened LD density in the R4 region postingestion, most LDs were smaller in size compared to those typically found in R2 (Figure 6A). On the other hand, the R5 midgut region appeared to be unaffected by the virus-induced changes in LD profiles.

These differential region-specific responses emphasize the complex interplay between SARS-CoV-2 and lipid metabolism, which could impact the outcome of viral replication.

### Plitidepsin mitigates SARS-CoV-2-induced pathogenesis in the midgut

**Figure 7:**
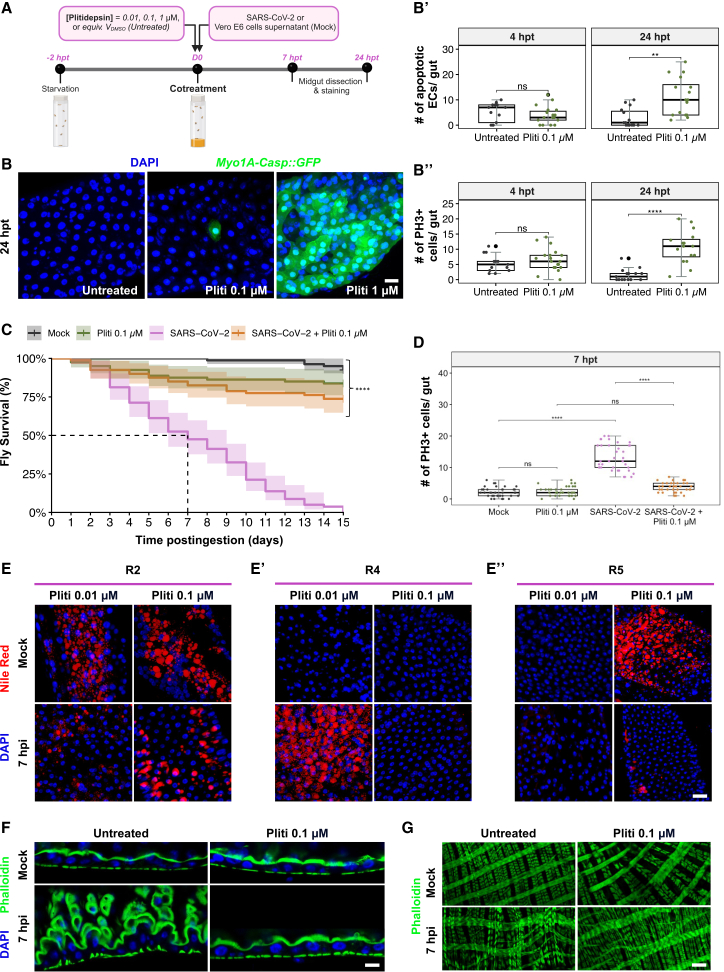
Plitidepsin mitigates SARS-CoV-2 activity in the midgut (A) Diagram summarizes the cotreatment procedure of flies with different doses of Plitidepsin and SARS-CoV-2. Created with BioRender. (B) Representative images of R4 midgut region, showing Caspase 3 activity (GFP) driven by *Myo1A-GAL4* in ECs at 24 h postingestion of 0.1 and 1 μM plitidepsin compared to untreated control. (B′) Quantification of apoptotic ECs (*Myo1A-Casp::GFP*), and (B″) mitotic ISCs (PH3-positive cells) in untreated guts or treated with 0.1 μM plitidepsin at 4 and 24 h posttreatment. Data were collected from three independent replicates with 6 midguts each (*n* = 18 midguts/condition/timepoint). *p*-values from one-way ANOVA are ns > 0.05, ∗∗ <0.01, and ∗∗∗∗ <0.0001. (C) Kaplan-Meier survival curves compare flies (*w*^*1118*^ genetic background) orally infected with SARS-CoV-2 or cotreated with 0.1 μM Plitidepsin, to mock flies that ingested Vero E6 cell supernatants or received 0.1 μM Plitidepsin alone. Data represent three biological replicates (*n* = 20 flies/condition/replicate). Shaded areas represent the 95% confidence intervals (CI). Statistical significance was measured using the Log rank test (*p* < 0.01). (D) Quantification of mitotic ISCs (PH3-positive cells) in infected gut with SARS-CoV-2 or cotreated with 0.1 μM Plitidepsin, compared to mock flies that ingested Vero E6 cell supernatants or received 0.1 μM Plitidepsin alone. Data were collected from three independent replicates, with 12 midguts analyzed per condition in each replicate (*n* = 36 midguts per condition). Statistical significance was assessed using one-way ANOVA. *p*-values: ns > 0.05, ∗∗∗∗ <0.0001. (E) Representative images show the distribution of lipid droplets at 7hpi in the anterior R2, (E′) in the posterior R4 and (E″) R5 regions, stained with Nile Red (red), following cotreatment with 0.01 μM and 0.1 μM Plitidepsin. The corresponding untreated control midguts are shown in Figure 6. 20 midguts were scored for this experiment. (F) Representative confocal sections of midguts show the brush border microvilli at 7hpi following cotreatment 0.1 μM Plitidepsin, compared to untreated guts (*n* = 10/condition/3 biological replicates). F-actin is stained using Phalloidin (green). DAPI is blue. Scale bars represent 30 μm. (G) Visceral muscle fibers in R4 at 7hpi, stained with Phalloidin (green) following cotreatment with 0, or 0.1 μM of Plitidepsin, compared to noninfected guts (*n* = 10/condition/3 biological replicates).

To explore potential antivirals effective against SARS-CoV-2-induced pathogenesis in the *Drosophila* midgut, we investigated the efficacy of Plitidepsin, a marine-derived cyclic depsipeptide isolated from the ascidian *Aplidium albicans*. Plitidepsin has shown potent preclinical efficacy against SARS-CoV-2 and has progressed to phase III clinical trials.^25^^,^^38^ This drug was co-administered orally at different doses (0.01 μM, 0.1 μM, and 1 μM), both with and without the viral solution (Figure 7A). Initially, to establish the appropriate dose range of Plitidepsin for subsequent experiments, we evaluated its toxicity *in vivo*. Employing the *Myo1A>Casp::GFP* transgenic line, we observed a dose-dependent increase in apoptotic EC counts at 24 h posttreatment (hpt), with a slight increase at 0.1 μM, which became excessive at 1 μM, indicating the high toxicity of this latter dose (Figures 7B and 7B′). We did not proceed to EC counts at 1 μM as the whole midgut becomes GFP positive. Similarly, we noted a significant dose-dependent increase in ISC proliferation, as evidenced by the rising number of PH3+ cells, quantified in Figure 7B”.

Based on these results, the 1 μM dose was excluded from experiments due to its toxicity, and only non-toxic concentrations were used in subsequent analyses. Notably, treatment with 0.1 μM Plitidepsin significantly attenuated the SARS-CoV-2-induced lethality in flies (Figure 7C), supporting the efficacy of this dose in mitigating virus-induced pathogenesis.

We then assessed the effect of Plitidepsin cotreatment on specific intestinal alterations induced in the early phase (7 hpi) of SARS-CoV-2 exposure, including ISC proliferation (Figure 7D), region-specific LD profiles (Figures 7E–7E″), epithelial cell multilayering (Figure 7F), and disturbances in visceral muscle fiber thickening (Figure 7G; as described earlier in Figures 6A, 3E, and 2E respectively). At the cellular level, cotreatment with 0.1 μM Plitidepsin effectively counteracted the virus-induced rise in ISC proliferation (Figure 7D). At the metabolic level, 0.01 μM Plitidepsin had no observable impact on LD alterations caused by SARS-CoV-2 (Figures 7E–7E″ and S7A, controls in Figure 6); however, a 0.1 μM dose effectively restored LD distribution to that observed in controls flies in both R2 and R4 regions. Plitidepsin ingestion alone did not alter LD distribution in these midgut regions, regardless of the dose (Figures 7E, 7E′, S7A, and 6). Interestingly, a 0.1 μM dose of Plitidepsin resulted in LD accumulation in the R5 region under non-infected conditions. We then investigated the potential of Plitidepsin in restoring the intricate architecture of the *Drosophila* intestinal epithelium caused by SARS-CoV-2. At 0.01 μM, Plitidepsin began to counteract the virus-induced epithelial damage, partially restoring epithelial integrity. Importantly, a 0.1 μM dose completely rescued the disrupted phenotype (Figure 7F). Nevertheless, Plitidepsin failed to exert any discernible rescue effect on the spasmodic status of visceral muscles at 7 hpi (Figure 7G). Intriguingly, Plitidepsin administration alone at 0.1 μM dose appeared to induce disrupted structuring of the visceral muscles all along the midgut compared to control conditions (Figure 7G).

These data provide supporting evidence for the therapeutic potential of Plitidepsin against SARS-CoV-2–induced pathogenesis *in vivo*, contributing to the growing body of research aimed at identifying effective therapeutics for combating COVID-19 and other viral outbreaks.

### Plitidepsin exhibits anti-SARS-CoV-2 activity and modulates lipid droplet accumulation in A549-ACE2 human pulmonary cells

Having demonstrated the modulatory effect of Plitidepsin on LD homeostasis in *Drosophila* midgut challenged with SARS-CoV-2 (Figures 7E and 7E′), we aimed to assess whether this metabo-modulating activity is conserved in a well-established human alveolar cell line that highly expresses ACE2 (A549-ACE2) for studying SARS-CoV-2 replication.

First, we confirmed that A549-ACE2 cells are permissive to the SARS-CoV-2 variant used in this study at a multiplicity of infection (MOI) of 0.1 (Figure S7B). Prior to assessing the antiviral effects of Plitidepsin in A549-ACE2 cells, we examined its cytotoxicity using an ATP assay at 24- and 48 hpt, with concentration inhibiting 50% of cell viability (CC_50_) values of 0.88 and 0.02 μM, respectively (Figure S7C). This allowed us to evaluate the antiviral effect of Plitidepsin in these cells by flow cytometry, resulting in 50% inhibition concentration (IC_50_) of 0.003 μM at 24 hpi (Figure S7D). We further validated this antiviral effect by immunofluorescence assay (Figure S7E) using an anti-Spike antibody. Based on these results, we decided to carry out our subsequent experiments at an MOI of 0.1 and at a Plitidepsin concentration of 0.01 and 0.1 μM, which are consistent with the doses used in the *Drosophila* experiments.

Strikingly, as observed in the R5 midgut region (Figure 7E”), our data indicated that Plitidepsin induces LD formation in non-infected A549-ACE2 cells in a dose-dependent manner, while reducing LD accumulation induced by SARS-CoV-2 at 24 hpi, as evidenced by Bodipy FLC12 LD-specific staining (Figures S7F and S7F′). This antiviral activity is consistent with our data obtained in the *Drosophila* R2 and R4 midgut regions (Figures 7E and 7E′).

Altogether, our findings in both the *Drosophila* midgut and human alveolar cells reveal a potential mechanistic link between Plitidepsin and LD homeostasis, highlighting the valuable use of the *Drosophila* midgut for unraveling region-specific metabolic modulation mediated by viral challenge.

## Discussion

Although respiratory symptoms dominate the clinical presentation of COVID-19, increasing evidence highlights GI involvement, particularly in patients reporting digestive complaints. Imaging studies have identified COVID-19-associated gut abnormalities, including bowel wall thickening, pneumoperitoneum, and intestinal perforations.^39^ Histopathological analyses of intestinal biopsies have further revealed epithelial alterations, such as cytoplasmic blebbing and cellular crowding in infected areas.^40^ These observations underscore the need for tractable *in vivo* models to investigate the mechanisms of SARS-CoV-2-induced intestinal pathogenesis.

**Figure 8:**
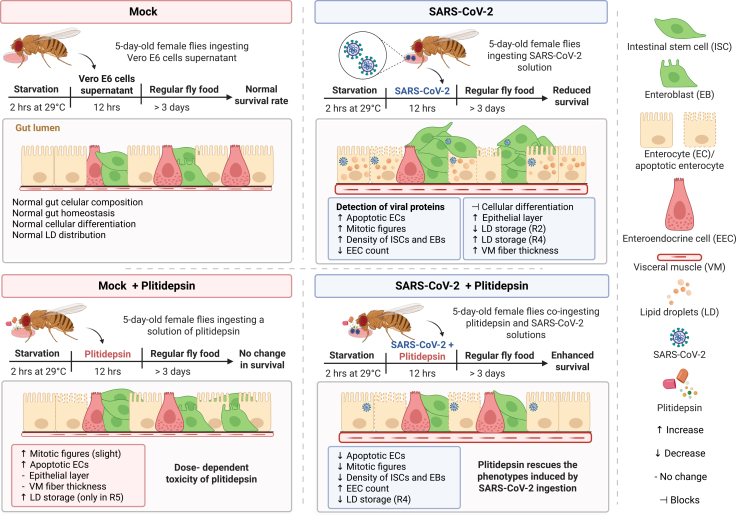
Summary of SARS-CoV-2 ingestion effects and Plitidepsin treatment on *Drosophila* midgut homeostasis Flies were orally exposed to SARS-CoV-2 or mock solutions, with or without the antiviral compound Plitidepsin. SARS-CoV-2 ingestion induced epithelial damage in the midgut, characterized by increased apoptosis of enterocytes, elevated mitotic activity, expansion of intestinal stem cells and enteroblasts, and thickening of the visceral muscle layer, ultimately reducing fly survival. Co-treatment with Plitidepsin mitigated most of these pathological effects, restoring gut morphology, limiting epithelial apoptosis and ISC proliferation, and re-establishing lipid droplet homeostasis.

Leveraging the well-characterized *Drosophila melanogaster* midgut system,^41^ our study provides a multiscale *in vivo* dissection of the gut alterations triggered by SARS-CoV-2 (Figure 8). We demonstrate that oral exposure to SARS-CoV-2 can initiate a localized and functionally relevant viral replication activity in the fly midgut. Immunofluorescence staining using antibodies against the viral Spike protein and the replication intermediate marker J2 (dsRNA-specific) revealed intracellular viral signals within midgut epithelial cells. These signals were absent during the initial ingestion phase (1–3 hpi), supporting the interpretation that the detected Spike signal reflects active intracellular replication rather than the passive retention of viral particles in the gut lumen. Despite the relatively low ingestion capacity of flies and the modest number of infectious particles recovered by plaque assay, viral RNA was consistently detected in midgut samples across multiple timepoints by RT-qPCR. This stable signal, together with immunostaining showing that up to 25% of epithelial cells display intracellular viral markers, indicates that SARS-CoV-2 is able to enter and initiate replication in the fly midgut epithelium. While this replication may be partial or abortive, it appears sufficient to disrupt epithelial homeostasis. Moreover, the recovery of infectious virus from fly homogenates on Vero cells, despite technical limitations, suggests that productive replication can occur in at least a subset of cells, though likely at low efficiency.

Remarkably, this limited exposure was sufficient to induce rapid and severe intestinal remodeling. Within 24 hpi, flies exhibited structural abnormalities including gut shortening, epithelial and visceral muscle thickening, and luminal food retention. These phenotypes progressively worsened and were associated with fly death, likely driven by gut dysfunction and potentially exacerbated by systemic effects. These early-onset changes seem to reflect an acute epithelial stress response involving both morphological remodeling and physiological disruption. Concomitantly, a plausible explanation for the altered phenol red signal in infected guts is a change in gut pH rather than impaired dye ingestion. In our assays, red dye was clearly visible in the abdomen within minutes of exposure in both control and virus-challenged flies, and only flies with robust abdominal dye uptake were selected for analysis, ensuring that pH measurements were performed on animals that had efficiently ingested the indicator. The paler or shifted coloration observed in challenged midguts is therefore best interpreted as a consequence of altered luminal acidity. Consistently, GO term analysis of downregulated genes at 12 hpi (Table S4) reveals enrichment for categories related to proton transport and associated processes, suggesting that SARS-CoV-2 ingestion perturbs molecular pathways controlling gut acid-base homeostasis. Together, these functional and transcriptional data support the notion that viral challenge disrupts gut physiology at least in part through the modulation of pH-regulating mechanisms.

At the cellular level, the emergence of a disorganized, multilayered epithelium, accumulation of apoptotic ECs, expansion of proliferative progenitors, and reduction of EECs all point to a rapid loss of epithelial homeostasis. This cellular destabilization appears to be part of a compensatory regenerative program. As early as 4 hpi, we observed a significant increase in EC apoptosis using the *Myo1A>Casp::GFP* reporter. Lineage-tracing with the *Su(H)-ReDDM* system revealed accelerated differentiation of ECs from the EB pool, consistent with a rapid repair response to acute epithelial injury. In contrast, *esg-ReDDM* showed no net change in progeny numbers, likely reflecting a balance between enhanced EC production and concurrent EEC loss at this timepoint, confirmed by the quantification of Prospero-positive cells. Although our study evaluated EC death, we cannot rule out EEC apoptosis. Attempts to assess EEC-specific death using *Prospero>Casp::GFP* proved inconclusive, likely due to the small size of EECs and difficulty distinguishing them from debris. We acknowledge this as a technical limitation and point to the need for additional approaches to dissect EEC turnover under viral challenge.

While the early response appears compensatory, our lineage-tracing data at 48 hpi reveal a more complex scenario. At this timepoint, all three systems, *esg-ReDDM*, *Su(H)-ReDDM* and *Delta-ReDDM*, show increased progenitor numbers, confirming sustained stem cell activation. Nevertheless, this expansion is not paralleled by efficient differentiation into mature intestinal cell types. In both *esg-ReDDM* and *Su(H)-ReDDM*, EBs appeared unable to fully commit to EC fates. However, the increase in *Delta-ReDDM* progeny likely reflects EB pool expansion, in line with *Su(H)-ReDDM* data. These observations suggest that SARS-CoV-2 sustains ISC proliferation while disrupting lineage differentiation, in addition to inducing cell death.

Even though viral RNA and replication markers became hardly detectable beyond 3 dpi, we observed a progressive increase in mortality, reaching nearly 50% by 7 dpi. This lethality likely reflects the physiological consequences of early viral exposure rather than ongoing viral replication. Consistently, the increased GFP-positive intestinal progenitor cells at 7 dpi indicate sustained stem cell activation. Such persistent regenerative activity suggests that the gut epithelium remains under stress, potentially leading to tissue exhaustion and systemic dysfunctions. While we cannot exclude the contribution of other factors, such as behavioral, neuronal alterations or metabolic imbalance, the observed phenotypes are more consistent with a post-viral inflammatory or stress-like state than with persistent infection.

This decoupling between proliferation and differentiation is not typically observed in bacterial infections,^42^ which generally induce transient ISC activation followed by prompt epithelial restoration.^43^ In contrast, our data support a model in which SARS-CoV-2 not only initiates epithelial turnover but also interferes with the proper execution of regenerative programs. This could reflect a general disruption of the differentiation machinery. Alternatively, it may represent a viral strategy to maintain a less immunocompetent, progenitor-rich environment. Importantly, such differentiation bottlenecks likely contribute to the epithelial disorganization and homeostatic collapse observed in challenged midguts.

Although we did not directly address the molecular mechanisms underlying SARS-CoV-2 entry or identify the specific midgut cell types permissive to viral replication, our findings lay important groundwork for addressing these questions. *Drosophila* harbors six conserved *ACE*-like genes,^44^ at least two of which, Ance and Acer, are expressed in the midgut (from flygut-seq database and^45^). These proteins have been proposed as potential orthologs of mammalian ACE2, the primary receptor for SARS-CoV-2. By integrating genetic tools and live imaging techniques available in *Drosophila*, further investigations could provide valuable insights into how SARS-CoV-2 interacts with the gut epithelial cells at the entry level.

In addition to structural and cellular remodeling, SARS-CoV-2 also induced early and significant midgut transcriptional reprogramming. Our transcriptomic profiling revealed that SARS-CoV-2 modulates the expression of a limited subset of genes in the midgut, suggesting a targeted rather than a broad response. The biphasic nature of the intestinal transcriptomic response is particularly noteworthy. The early activation phase is characterized by the predominance of upregulated genes (e.g., lipid metabolism, autophagy, and stress-related genes) and coincides with the onset of detectable viral replication, likely reflecting an immediate attempt by the host to counteract the infection. In contrast, the subsequent downregulation of transcriptional activity, dampening key cellular processes, may denote an adaptive mechanism aiming to mitigate prolonged stress or defense responses. Canonical immune genes were not among the dominant signatures, suggesting that the host response to SARS-CoV-2 in the fly midgut primarily involves metabolic and homeostatic pathways rather than classical antiviral immunity.

Interestingly, challenged midguts revealed a pronounced upregulation of genes involved in lipid metabolism as early as 4 hpi, including those related to fatty acid β-oxidation, phospholipid remodeling, and LD dynamics. GO enrichment further highlighted deregulation in pathways such as glycerophospholipid metabolism and fatty acid elongation, suggesting that the enteric epithelium rapidly undergoes metabolic adaptation upon viral exposure. To assess the spatial and functional relevance of these transcriptional changes, we performed *in vivo* lipid staining using both Nile Red and BODIPY FL C12. Under mock conditions, LDs were largely restricted to anterior midgut regions. Upon viral exposure, however, we observed a striking reduction of LD in the R2 region and its accumulation in R4. These observations suggest that lipid metabolism is not only transcriptionally reprogrammed but also spatially reorganized, possibly as a result of viral manipulation or host compensatory responses. LDs are dynamic cellular storage organelles composed of neutral lipids, mainly triglycerides and cholesterol esters, enclosed within a phospholipid monolayer associated with various proteins.^35^ While traditionally viewed as energy reservoirs, LDs also serve as hubs for lipid signaling and immune regulation and are increasingly recognized as key host platforms hijacked by intracellular pathogens. Viruses, in particular, have evolved strategies to exploit LDs as nutrient reservoirs or scaffolds for replication complexes.^46^ Some lipid-enveloped viruses such as CoVs and flaviviruses recruit LDs during their infection cycle by triggering their accumulation in host cells and modifying the lipid composition of these organelles to enhance viral replication.^46^ Hence, LDs could serve as a platform for the virus to acquire lipids and components necessary for the assembly of new viral particles.^47^ Viruses can also manipulate LDs for immune evasion by dissimulating within these particles to protect viral factors from recognition and degradation by the host immune systems.^46^ Nevertheless, infected cells may also use LDs as part of their antiviral defense strategies. For example, some LD proteins, such as Viperin, have been implicated in viral restriction by interfering with viral assembly or triggering a variety of antiviral responses.^48^ The differential regulation of LDs in the midgut could be linked to different metabolic functions or immune environments within the midgut. Functional interaction studies, along with the profiling of LDs and metabolic components across different gut regions, could provide insights into the metabolic requirements of SARS-CoV-2. Additionally, such studies may elucidate whether these antagonistic effects reflect a possible viral invasive strategy in the anterior midgut versus a LD-mediated cellular defense mechanism in the distal parts of the alimentary canal.

Comorbidities such as obesity and diabetes are known to exacerbate COVID-19 outcomes.^49^ These conditions are often associated with altered lipid metabolism and chronic inflammation, which could further influence the viral pathogenesis observed in the gut. Through a screening of metabolic genes,^50^ a recent study found that key players such as acetyl-CoA carboxylase-α (ACACA) and fatty acid synthase (FASN) can either inhibit or promote viral infection. Notably, the study highlighted the efficacy of Orlistat, an FDA-approved anti-obesity drug, as an inhibitor of FASN. Orlistat was shown to inhibit the *in vitro* replication of SARS-CoV-2 variants and significantly reduce viral levels and lung pathology in a mouse model of SARS-CoV-2 infection, improving survival rates. Future research using available fly models of obesity and diabetes could help elucidate how these comorbidities influence the gut’s response to SARS-CoV-2 and whether targeting lipid metabolism, as demonstrated with Orlistat, might provide therapeutic benefits for patients with these conditions. This could pave the way for repurposing lipid metabolism inhibitors for the prevention and treatment of severe COVID-19, warranting clinical trials to evaluate their efficacy in humans.

Studies on other repurposed drugs, such as hydroxychloroquine and remdesivir, revealed limited or no benefits in altering the disease progression.^51^ Importantly, Plitidepsin, initially developed for multiple myeloma, has shown potent preclinical activity against SARS-CoV-2.^25^ This drug, which inhibits the eEF1A translation cofactor,^25^ shows promise in both animal studies and early human trials,^38^ though its full toxicity profile is pending. Interestingly, our results show that Plitidepsin mitigated SARS-CoV-2–induced gut phenotypes, suggesting antiviral activity in this model system. Despite its cytotoxic potential at higher concentrations, the oral administration of non-toxic doses of Plitidepsin significantly improved fly survival in the context of SARS-CoV-2 challenge. This phenotypic rescue highlights the utility of the fly model in assessing functional outcomes of viral exposure and therapeutic interventions.

Beyond its impact on mortality, Plitidepsin treatment attenuated several early virus-induced phenotypes in the midgut. Notably, it restored epithelial architecture, reduced the overactivation of ISCs, and normalized the distribution of LDs, particularly in the R2 and R4 compartments where metabolic disruptions were most prominent. Although it does not restore the spasmodic visceral muscle phenotype, these effects suggest that Plitidepsin may limit virus-associated effects and/or modulate host responses to restore homeostatic balance in challenged tissues. Further investigation is necessary to determine whether Plitidepsin exerts its antiviral effects in the midgut through general translation inhibition or by regulating other cellular mechanisms, such as the modulation of lipid metabolism as observed in non-infected conditions, both in *Drosophila* and human alveolar cells.

In conclusion, our study features the multifaceted impact of SARS-CoV-2 on the GI system, utilizing the *Drosophila melanogaster* model to reveal critical aspects of virus-induced pathogenesis and host response. The findings emphasize the complexity of viral interactions with gut homeostasis, particularly in relation to tissue renewal and lipid metabolism. Additionally, our results demonstrate that *Drosophila* serves as a valuable model for *in vivo* screening of new bioactive compounds to combat COVID-19. Specifically, our study supports the potential of Plitidepsin as a therapeutic agent against SARS-CoV-2, warranting further examination of its mechanism of action and therapeutic applications. Finally, given the continuous emergence of new SARS-CoV-2 variants, our research suggests that *Drosophila* can be invaluable in understanding how viral mutations impact intestinal pathogenesis. Together, our findings not only uncover new insights into SARS-CoV-2 gut pathogenesis but also establish a versatile model to accelerate antiviral discovery and dissect host-virus interactions at epithelial interfaces.

### Limitations of the study

While this work establishes *Drosophila melanogaster* as a tractable *in vivo* model for SARS-CoV-2-induced intestinal pathogenesis, several limitations should be considered. First, the oral infection protocol used here does not fully mirror natural exposure routes in humans, and viral uptake may vary slightly between individual flies; moreover, *Drosophila* is not a natural host for SARS-CoV-2, resulting in limited and potentially abortive viral replication. Second, although we observed extra-intestinal effects, including alterations in ovaries and rectal tissue, the systemic consequences of viral ingestion were not comprehensively examined. Third, our study focused on a single SARS-CoV-2 lineage (D614G), and the pathogenic potential of other variants with strong GI tropism, such as Delta or Omicron, remains unexplored in this model. Fourth, transcriptomic analyses were performed on whole midgut preparations, which may mask region-specific responses, as suggested by the spatially distinct LD alterations observed along the gut; higher-resolution or cell-type-specific profiling would refine these insights. Finally, functional validation of metabolic pathways, using mutants affecting lipid, glucose, or protein metabolism, was beyond the scope of this study but will be essential to mechanistically link metabolic rewiring to viral pathology. Addressing these limitations in future work will strengthen the translational relevance of the *Drosophila* midgut model for investigating SARS-CoV-2 intestinal pathogenesis and antiviral interventions.

## Resource availability

### Lead contact

Requests for further information and resources should be directed to and will be fulfilled by the lead contact, Dani OSMAN (dani.osman@univ-reunion.fr)mailto.

### Materials availability

This study did not generate new unique reagents. All analyses and data generated in this study are available from the lead contact without restriction.

### Data and code availability

• Data: Raw sequence data have been deposited at the Sequence Read Archive (SRA PRJNA1137395 for *Ecc15* and SRA PRJNA1137405 for SARS-CoV-2) and are publicly available. This study does not involve a clinical trial; thus, no registration or associated links apply. Any additional information required to reanalyze the data reported in this paper is available from the lead contact upon request.
• Code: This study did not generate custom code. Any scripts used for standard data processing and analysis are available from the lead contact upon reasonable request.
• Other items: Any additional information required to reanalyze the data reported in this paper is available from the lead contact upon request. Details of reagents, datasets, and software used in this study are provided in the key resources table.

## Acknowledgments

We thank Armel Gallet, Marie-Lise Gougeon, Philippe Despres, and Radwan Kassir for their valuable insights on this research. We are grateful to Cyrille Lebon and Hervé Pascalis for ensuring optimal experimental conditions at the PLATIN-OI BSL3 platform. Library sequencing was carried out at Cornell University BRC Genomics Core Facility (RRID:SCR_021727). This study was funded by POE FEDER 2014-20 of the Conseil Regional de La Reunion (TFORCE-COVIR, N°20201437-0027601), and Research Federation BioST, La Reunion University (COVIPID). LEK received doctoral fellowships from the National Council for Scientific Research of Lebanon (CNRS-L), the Lebanese University and ERASMUS+MIC program. Buchon's laboratory was supported by 10.13039/100000002NIH R01AI148529, R01AI148541, and 10.13039/100000001NSF IOS2024252.

## Author contributions

Project Setup: L.EK., P.M., C.EK., and D.O.; methodology and validation: L.EK., C.EK., and D.O.; investigation: L.EK., J.G., C.EK., and D.O.; RNA-seq library preparation and Raw data curation: P.N. and N.B.; RNA-seq formal analysis: L.EK., P.N., N.B., C.EK., and D.O.; writing – original draft: L.EK. and D.O.; writing, review, and editing: all authors. supervision and Funding Acquisition: P.M., C.EK., and D.O. conceptualization and project administration: C.EK. and D.O.

## Declaration of interests

The authors declare no competing interests.

## Declaration of generative AI and AI-assisted technologies in the writing process

The authors declare no AI was used to generate or evaluate data or to write the manuscript.

## STAR★Methods

### Key resources table

**Table:** 

REAGENT or RESOURCE	SOURCE	IDENTIFIER
**Antibodies**		

J2 dsRNA antibody	Merck Millipore	MABE1134; RRID: AB_2819101
Mouse anti-Prospero antibody (1:100)	DSHB	(MR1A)-c; RRID: AB_528440
Rabbit polyclonal Anti-phospho-Histone H3 (Ser10) (1:1,000)	Millipore	06-570; RRID: AB_310177
Anti-CoV2RBD-c1-hIgG1 (1:1000)	Invivogen	CoV2rbdc1-mab1
Alexa Fluor 488 goat anti-Human IgG (1:1000)	Invitrogen	A11013; RRID: AB_2534080
Alexa Fluor 594 donkey anti-Rabbit IgG (1:1000)	Invitrogen	A21207
Alexa Fluor 488 goat anti-Mouse IgG (1:1000)	Invitrogen	A11029; RRID: AB_2534088
Alexa Fluor 647 goat anti-Mouse IgG (1:1000)	Invitrogen	A-21235; RRID: AB_2535804
Alexa Fluor 594 goat anti-Human IgG (1:1000)	Invitrogen	A11014

**Bacterial and virus strains**		

SARS-CoV-2	Available in the lab, sequence available on GISAID	RUN-PIMIT8

**Chemicals, peptides, and recombinant proteins**		

Amphotericin B	PAN Biotech	P06-01100
Carboxymethylcellulose	Sigma	C5013-500g
Crystal Violet	Sigma Aldrich	C0775-100G
Ethanol	Sigma Aldrich	24103-1L-R
DAPI (4’,6-diamidino-é-phenylindole)	Thermo Fischer Scientific	
16% Paraformaldehyde, methanol free	Thermo scientific	Cat# 043368.9M
Formaldehyde 36% in aqueous solution	VWR	20909.290
Fetal Bovine Serum	PAN Biotech	P40-37500
Eagle Medium (MEM)	PAN Biotech	P04-08500
DMEM	PAN Biotech	P04-03600
DMSO	Sigma	D4540
Glycerol	Sigma	G7893
Isopropanol	Sigma	563935
Stable Glutamine	PAN Biotech	P04-82100
Nile Red	Sigma Aldrich	72485-100mg
Bodipy^TM^ FL C12 (low-density) lipoprotein (LDL)	Invitrogen	L3483
PBS	Corning	20-031-CV
Phalloidin iFluor 488 Conjugate (1:1000)	Abcam	Ab176753
Phalloidin iFluor 594 Conjugate (1:1000)	Abcam	Ab176757
Phenol Red	Sigma	N/A
Penicillin Streptomycin	PAN Biotech	P06-07100
Plitidepsin	Pharmamar	PM90001
Puromycin	Invivogen	Ant-pr
RNeasy Mini Kit	Qiagen	74106
Random Primer	Promega	C1181729819601
Bovine Serum Albumin	Duscher	PAO228HO2A
Sodium Pyruvate 100mM	Dutscher	L0642-100
Superscript III	Invitrogen	18080-093
SYBR Green master mix	Thermo Fischer Scientific	
Triton X100	Sigma Aldrich	T8787-50mL
Trypsin	PAN Biotech	P10-022-100

**Critical commercial assays**		

RNeasy Mini Kit	Qiagen	74104

**Deposited data**		

RNA sequencing data for Ecc15		SRA PRJNA1137395
RNA sequencing data for SARS-CoV-2		SRA PRJNA1137405

**Experimental models: Cell lines**		

Vero E6	ATCC	CRL1586
A549-ACE2	Invivogen	a549-ace2

**Experimental models: Organisms/strains**		

*w*^*1118*^	BDSC	N/A
*w; esg-GAL4,UAS-GFP,tubP-GAL80*^*ts*^	Jiang et al.^52^	*esg*^*ts*^*::GFP*
*w; tubP-GAL80*^*ts*^; *DL-GAL4,UAS-GFP/TM6b*	Zeng et al.^53^	*Delta*^*ts*^*::GFP*
*w; Myo1A-GAL4/SM6b; UAS-GC3*^*AiG7s*^*/TM6b*	Jneid et al. and Schott et al.^31^^,^^32^	*Myo1A-Casp::GFP*
w; *UAS-GFP::CD8; UAS-H2B::RFP/TM2*	Antonello et al.^34^	*ReDDM*
*w; Su(H)-GBE-GAL4, UAS-CD8::GFP/CyO; UAS-H2B::RFP, tubP-GAL80*^*ts*^*/TM6C*	Zeng et al.^53^	*Su(H)-ReDDM*

**Oligonucleotides**		

Primer: Fwd_RpL32 ATGCTAAGCTGTCGCACAAATG	Available in the lab	N/A
Primer: Rev_RpL32 GTTCGATCCGTAACCGATGT	Available in the lab	N/A
Primer: Fwd_HKUN TAATCAGACAAGGAACTGATTA	Available in the lab	N/A
Primer: Rev_HKUN CGAAGGTGTGACTTCCATG	Available in the lab	N/A
Primer: Fwd_ORF1ab CTAGGACCTCTTTCTGCTCA	Available in the lab	N/A
Primer: Rev_ORF1ab ACACTCTCCTAGCACCATCA	Available in the lab	N/A

**Software and algorithms**		

CytExpert software	Beckman Coulter, Brea, CA, USA	Version 2.1.0.92
ImageJ 1.52a	Rasband, W.S., U. S. NIH, Bethesda	https://imagej.net/
Graphpad Prism		https://www.graphpad.com/
R Statistical Software v4.2.0	The R foundation for statistical computing	https://www.r-project.org; RRID: SCR_001905
ZEN Digital Imaging for Light Microscopy	Carl Zeiss	https://www.zeiss.com/microscopy/int/downloads.html RRID:SCR_013672
ZENLite	Carl Zeiss	https://www.zeiss.com/microscopy/en/products/software/zeiss-zen-lite.html RRID:SCR_023747
NiKon C2Si	Nikon	https://cmm.centre.uq.edu.au/nikon-c2si-upright-confocal-microscope
BioRender		https://www.biorender.com/

**Other**		

Nutri-fly	Bloomington	66-112

### Experimental model and study participant details

#### Cell lines

The African green monkey (*Chlorocebus sabaeus*) kidney epithelial cell line Vero E6 (ATCC, CRL-1586) was used for SARS-CoV-2 isolation, propagation, and titration. Lung carcinoma cell lines A549-ACE2 (Invivogen) are genetically engineered cells to stably express human ACE2, that are derived from A549 cells. They were used to test the toxicity, antiviral activity of plitidepsin, *in cellula.* Vero E6 cells originate from a male animal; A549 cells originally derive from 58-year-old male human patient with lung carcinoma. Sex-specific biological variables are therefore not applicable to experimental comparisons in this study. Cell line authentication was performed by the supplier. No additional authentication was performed in-house. Cells were handled under biosafety level 3 (BSL-3) conditions.

#### SARS-CoV-2 strain

The SARS-CoV-2 strain used in this study was isolated in collaboration with the Centre Hospitalier Universitaire (CHU, La Réunion) in April 2020 from a nasopharyngeal swab obtained from a SARS-CoV-2–positive individual visiting La Réunion Island. Biological material was stored using standard diagnostic procedures. No personal data were collected or analysed, and the sample is fully anonymized. In accordance with national regulations and institutional guidelines for Good Scientific Practice, ethical committee approval and the requirement for informed consent were waived. Accordingly, written informed consent from the participant was not required under applicable national legislation and institutional policies. Viral genome sequencing identified the isolate as belonging to the ancestral European D614G variant. Full genome sequences are available through the GISAID database under accession ID RUN-PIMIT8. All work involving infectious virus was conducted in a certified BSL-3 laboratory in accordance with institutional and national biosafety regulations.

#### Drosophila melanogaster

*In vivo* experiments were performed using only *Drosophila melanogaster* species. Only adult female flies were used in all assays to minimize variability associated with sex-specific differences in feeding behaviour and intestinal physiology. Flies were used at 3–7 days post-eclosion, corresponding to young adult stages. They were randomly allocated to experimental conditions following eclosion. As *Drosophila melanogaster* is an invertebrate model, no institutional animal ethics approval was required for this study. Fly rearing and husbandry were performed and maintained in a level 1 insectarium, SARS-CoV-2 exposure experiments were conducted in a certified BSL-3 laboratory in accordance with institutional and national biosafety regulations.

### Method details

#### SARS-CoV-2 clinical strain isolation

A SARS-CoV-2 strain was isolated from a nasopharyngeal swab taken from a tourist visiting La Reunion Island in April 2020. The individual had been confirmed positive for SARS-CoV-2 through RT-qPCR testing. The isolation process followed the methodology previously described by.^27^ In brief, the African green monkey kidney cell line, Vero E6 (ATCC-CRL1586), was cultured at 37°C in a 5% CO_2_ atmosphere within a 25 cm^2^ flask. The flask was filled with 5 ml of a solution containing Eagle’s Minimum Essential Medium (MEM, PAN Biotech, P04-08500) enriched with 5% heat-inactivated fetal bovine serum (FBS, PAN Biotech, P40-37500), 2 mmol/L L-glutamine, 1 mmol/L sodium pyruvate, 100 U/mL penicillin, 100 μg/mL streptomycin, and 0.5 μg/mL Amphotericin B (PAN Biotech, Aidenbach, Germany). One day later, the cells were washed with MEM containing 2% FBS, followed by the inoculation with 75 μl of the SARS-CoV-2 swab specimen. After a two-hour incubation, the inoculum was removed, cells were washed again, and replaced with fresh MEM 2% FBS. The flasks were then incubated and monitored daily for any cytopathic effects. Three days postinoculation, supernatants were collected, centrifuged at 400 x g for 5 minutes at 4°C to remove cell debris, aliquoted, and then stored at -80°C.

The viral genome extracted from these samples was sequenced, with >90% coverage, using the MinION sequencing nanopore technology. The sequencing results confirmed the strain as belonging to the ancestral European D614G variant. Full genomic data have been made available on GISAID under the accession ID: RUN-PIMIT8. All experiments involving SARS-CoV-2 were conducted in a Biosafety Level 3 Laboratory (BSL-3) to ensure stringent containment and safety measures.

#### SARS-CoV-2 production and titration

To produce a high-titer virus stock solution, 200 μL of the isolated SARS-CoV-2 was spread in permissive Vero E6 cells within 75 cm^2^ flasks using MEM supplemented with 2% FBS. Cytopathic effect indicative of viral infection was observed three days postinoculation, time at which viral lysates were collected. In parallel, supernatants from uninfected Vero E6 cells were harvested to serve as a mock solution. Following the protocol outlined previously, both types of supernatants were centrifuged, aliquoted, and stored at -80°C.

The concentration of viral progeny was determined using the PFU assay. For the assay, Vero E6 cells were plated in 24-well plates at a density of 70,000 cells per well. The next day, the cell monolayers were infected with 100 μL of a serial tenfold dilution of the SARS-CoV-2 solution, with the mock solution tested in parallel as a control. Two hours after allowing the virus entry, the cells were overlaid with 1% carboxymethyl cellulose (CMC, Sigma, C5013-500g) in MEM 5% FBS to restrict virus spread to directly adjacent cells. After incubating for three days at 37°C in a 5% CO_2_ atmosphere, the CMC overlay was removed. Cells were then washed twice with phosphate-buffered saline (PBS) and fixed with 3.7% paraformaldehyde (PFA, Thermo scientific, Cat# 043368.9M) in PBS for 20 minutes. Subsequent staining with 0.1% crystal violet (Sigma, C0775-100G) in 20% ethanol (Sigma, 24103-1L-R) allowed for visual identification of plaques as clear zones against a violet background of uninfected cells. After rinsing the plates with water to remove excess stain, plaques were counted and expressed as PFU/mL. All titrations were performed in duplicates. The viral titer was calculated using the formula: Titer (PFU/mL) = number of plaques counted x dilution factor/ volume of virus solution added to the wells (in mL).

To carry out PFU counts on flies, Vero E6 cells were infected with 100 μL of fly lysates diluted in MEM 2% FBS. Titration assays were conducted with at least five different replicates per experiment, and three independent biological replicates were performed. It was crucial not to fix the flies or midguts in paraformaldehyde prior to homogenization for this assay.

#### Fly strains and crosses

The fly strains used in this study: *w*^*1118*^ (BDSC, #5905), *w; esg-GAL4,UAS-GFP,tubP-GAL80*^*ts*^ (*esg*^*ts*^*::GFP,*^52^), *w; tubP-GAL80*^*ts*^; *DL-GAL4,UAS-GFP/TM6b* (*Delta*^*ts*^*::GFP*,^53^), *w; Myo1A-GAL4/SM6b; UAS-GC3*^*AiG7s*^*/TM6b* (*Myo1A-Casp::GFP,*^31^^,^^32^), *w; UAS-GFP::CD8; UAS-H2B::RFP/TM2* (*ReDDM,*^34^), *w; Su(H)-GBE-GAL4, UAS-CD8::GFP/CyO; UAS-H2B::RFP, tubP-GAL80*^*ts*^*/TM6C* (*Su(H)-ReDDM,*^53^). Fly stocks were maintained at 25°C in polystyrene vials containing a standard culture medium (Bloomington recipe media, Nutri-fly, Cat#: 66-112) complemented with Amphotericin B (0.5 μg/mL). The flies experienced a regular 12:12-hour light-dark cycle and were transferred to fresh food vials weekly.

For lineage tracing studies, virgin females from the *Delta*^*ts*^*-GFP* and *esg*^*ts*^*-GFP* were crossbred with *ReDDM* males, whereas *Su(H)-ReDDM* fly stock were crossed with males possessing *w*^*1118*^ genetic background. The different crosses were kept at 18°C. Subsequently, in all our *ReDDM* experiments, *Gal4* activity was temporally controlled using *tubP-GAL80*^*ts*^: the resulting F1 mated females were maintained at 18°C to keep *Gal4* repressed, then shifted to 29°C in adults for 2 days prior to viral ingestion to induce expression of mCD8::GFP (short-lived) and H2B::RFP (long-lived) in the chosen progenitor populations. This configuration was applied to several *Gal4* drivers, including esg-Gal4 as a canonical ISC/EB/preEEC driver. In this context, *esg-GAL4* is active in progenitors but is switched off upon differentiation, while the stable H2B::RFP protein is known to persist for at least 28 days in differentiated progeny, whereas mCD8::GFP is rapidly lost. Thus, RFP^+^/GFP^-^ cells correspond to differentiated cells derived from *esg*^*+*^ (or other progenitor-specific Gal4) lineages during the labelling window, rather than simply long-lived or non-dying cells.

#### Fly oral exposure and treatment

We first subjected female flies to a 2-hour starvation period at 29°C in empty vials. These vials were prepared with standard culture medium completely covered at the top with a Whatman paper disk. Specifically, a 22 mm diameter disk was used, onto which was added 150 μL of either a Vero E6 cell supernatant (serving as the control, or “Mock” group),a SARS-CoV-2 clinical isolate solution at 3.10^6^ PFU/mL, or a heat-inactivated viral solution, obtained by incubating the viral stock at 60°C for 30 min. Following a 12-hour exposure period to these conditions, the flies were transferred to new standard food vials daily.

To evaluate the antiviral effectiveness of Plitidepsin (Pharmamar, PM90001) *in vivo*, starved flies were fed a solution containing either the virus or the mock solution, supplemented with different Plitidepsin concentrations (0.01, 0.1, and 1 μM).

#### Fly survival assay

To evaluate the effects of oral SARS-CoV-2 and Plitidepsin treatment on Drosophila survival, flies were housed in groups of 20 per vial. For the infection assays presented in Figure 1, each experimental condition was represented by nine vials, and the experiment was independently replicated three times. For the Plitidepsin rescue experiments shown in Figure 7, each condition included three vials, with three independent biological replicates. Survival was monitored daily over a two-week period, and dead flies were removed and recorded throughout the experiment. To mitigate risks such as drowning in the food medium or fungal contamination, the vials were replaced daily.

#### RNA extraction and RT-qPCR

For RNA extraction and subsequent RT-qPCR analysis, samples consisting of either five whole flies or five dissected midguts (excluding the Malpighian tubules) were homogenized in 500 μL of MEM supplemented with 2% FBS. Homogenization was achieved using a Tissue Lyser II, through 3 cycles of 1 minute each at a frequency of 30 Hz. The homogenates were then centrifuged at 4°C for 10 minutes at maximum speed. From each sample, 250 μL of the supernatant was utilized for RNA extraction, with the remaining volume being stored at -80°C for PFU assays. Total RNA was extracted using the RNeasy Mini Kit (Qiagen, Valencia, CA, USA) in accordance with the manufacturer' instructions.

For cDNA synthesis, a two-step reverse transcription process was employed using ProtoScript II Reverse Transcriptase (Bioloabs) and Random Primer 6 (BioLabs). The reverse transcription conditions were as follows: initial denaturation at 70°C for 5 minutes, primer annealing at 25°C for 10 minutes, extension at 42°C for 50 minutes, and a final inactivation step at 65°C for 20 minutes. Quantitative real-time PCR (qPCR) targeting the Nucleoprotein (N) gene of SARS-CoV-2 was conducted using SYBR Green reagent (Thermo Fisher Scientific). Reaction mix included 12.5 μL of SYBR Green, 1 μL of each primer (1 μM), 5.5 μL of PCR water and 5 μL of cDNA. The qPCR assays were performed on a CFX96 Real-Time PCR Detection System (Bio-Rad), under the following conditions: activation at 95°C for 15 min and 40 cycles of denaturation and annealing/extension at 95°C for 15 s and 60°C for 1 min. Sequences of the primers used: Forward-HKUN 5’TAATCAGACAAGGAACTGATTA3’, Reverse-HKUN 5’CGAAGGTGTGACTTCCATG3’, Forward-ORF1ab 5’CTAGGACCTCTTTCTGCTCA3’, Reverse-ORF1ab 5’ACACTCTCCTAGCACCATCA3’. To establish a standard curve, cDNA synthesized from a SARS-CoV-2 positive sample previously verified in our laboratory was used as a positive control. Additionally, each sample was analyzed for the expression of the *Drosophila* housekeeping gene RpL32 to ensure accurate quantification. Sequences of RpL32 primers: Forward_RpL32 5’ATGCTAAGCTGTCGCACAAATG3’, Reverse_RpL32 5’GTTCGATCCGTAACCGATGT3’.

#### Immunostaining

For midgut study, prior to tissue dissection in a BSL-3 containment facility, flies were initially anesthetized by placing them on ice for 1 minute. They were then gently rinsed in 70% ethanol for 2 minutes to ensure sterility and minimize contamination risk. The guts and ovaries were dissected out and fixed in 4% paraformaldehyde for 30 minutes at room temperature. Tissues were subsequently permeabilized with 0.1% Triton X-100 in PBS (PBST) for 5 minutes. Samples were blocked using 1% Bovine Serum Albumin (BSA) in PBST for 30 minutes at room temperature. Primary antibody incubation was carried out overnight at 4°C in a solution of PBST with 1% BSA. The primary antibodies utilized included: mouse anti-Prospero (DSHB Cat# MR1A, diluted 1:500), rabbit anti-Phospho-Histone H3 (Millipore Cat# 06-570, diluted 1:1000), mouse anti-dsRNA clone rJ2 (Merck Millipore, Cat# MABE1134) diluted 1:60), and human anti-Spike protein (Invivogen Cat# cov2rbdc1-mab1, diluted 1:1000). Following the primary antibody incubation, samples underwent three washes in PBST for 5 minutes each, then were incubated for 2 hours at room temperature with Alexa Fluor-conjugated secondary antibodies (Invitrogen, A11013, A21207, A11029, A-21235, A11014, diluted 1:1000). For cytoskeletal visualization, F-actin in both gut and ovary samples was stained using iFluor-conjugated Phalloidin (Phalloidin iFluor 488 Conjugate (1:1000), Abcam Ab176753 and Phalloidin iFluor 594 Conjugate (1:1000), Abcam Ab176757). Nuclei were stained with DAPI (Sigma, at 0.5 mg/mL). After staining, tissues were washed three times with PBST and mounted on slides using a 1:1 mixture of PBS and glycerol. Gut samples were imaged using a NIKON C2si confocal microscope with NIS Elements software or Nikon Eclipse 80i fluorescence microscope, and ovary images were captured with a Nanozoomer S60 (Hamamatsu). All images were taken in the R4 midgut region, unless stated otherwise.

For *in cellulo* study, human alveolar cells overexpressing ACE2 (Invivogen, A549-ACE2) were regularly maintained in complete growth medium, supplemented with 0.5 μg/mL of Puromycin (Invivogen Ant-pr). Cells were plated on coverslips at a density of 100,000 cells/well. The following day, cells were subjected to either the mock, or the viral solution at different multiplicities of infection (MOI = 0.1, 0.2, 0.5, and 1). They were also cotreated with a ten-fold dilution series of Plitidepsin. 24 hours later, cells were rinsed with PBS, and fixed for 20 mins in PBS, PFA 3.7%. Next, cells were permeabilized with PBST for 5 mins, blocked with BSA 1% for 20 mins, and incubated then at room temperature with human anti-Spike protein (diluted 1:1000). Two hours later, cells were rinsed again with PBS and incubated for 30 additional minutes with Alexa Fluor 594 goat anti-Human IgG (diluted 1:1000). Nuclei were stained with DAPI. Two additional washes in PBS were performed and coverslips were mounted using 1:1 mixture of PBS and glycerol. Images were acquired using a NIKON C2si confocal microscope with NIS Elements software.

#### Fly- to- fly transmission assay

Horizontal transmission potential was evaluated by co-housing SARS-CoV-2–exposed flies with unexposed partners. W^-^ (white-eyed) flies were starved for 2 h and subsequently orally exposed to either SARS-CoV-2 or mock solutions for 12 hours. Following exposure, 20 challenged W^-^ flies were transferred to vials and co-housed with 10 unexposed W^+^ (red-eyed) flies. This setup allowed physical contact and shared feeding surfaces, mimicking potential routes of horizontal transmission. The control group (Mock) consisted of W^-^ flies exposed to Vero E6 cell supernatants. Flies were maintained under standard rearing conditions, and survival was monitored daily. Data were collected across four independent experiments, resulting in a total of 80 W^-^ and 40 W^+^ flies per condition.

#### Midgut morphometry

To assess midgut length and width, the guts were imaged using a Nikon SMZ18 stereoscope equipped with a Hamamatsu digital camera (Life Sciences, Japan). Length measurements were conducted by tracing a spline line along the midgut, from the center of the proventriculus to the junction between the midgut and hindgut, marked by the branching of the Malpighian tubules, whereas width was assessed by tracing a line throughout the R4 region. Measurements were taken for six guts per condition, and the process was repeated independently three times. For the observation of the crop and midgut lumen, a Nikon SMZ18 stereomicroscope equipped with a Hamamatsu digital camera was used (Life Sciences, Japan). Images were acquired from six samples per condition in each of the three experimental replicates. To measure muscle fiber thickness, the midgut was stained with Phalloidin, and the width of the longitudinal fibers was measured using ZenLite software. Measurements focused on sections from the posterior midgut in R4 region, with 10-13 fibers measured per gut across six guts for each condition.

#### Midgut cell quantification

Mitotic cells marked for PH3 staining and apoptotic enterocytes were counted across all gut regions. Prospero+ cells were specifically counted in the R4 region. The enumeration was conducted using a Nikon Eclipse 80i fluorescence microscope. *esg*^*+*^ cells (in *esg*^*ts*^*>GFP* line), *Delta*^*+*^ cells (in *Delta*^*ts*^*>GFP* line), as well as both GFP^+^ RFP^+^ and RFP^+^ only cells (in *ReDDM* lines) were enumerated within a designated section of R4. Cell densities were calculated by normalizing the cell counts to a 20,000 μm^2^ surface midgut area using Zen software (ZEN Digital Imaging for Light Microscopy). Data were collected from six midguts per condition for each replicate, and the independent experiments were conducted a minimum of three times to ensure reliability.

#### Fecal spot quantification

Intestinal transit was assessed using a bromophenol blue (BPB)-based excretion assay. BPB is an inexpensive, non-toxic, heat-stable dye that can be mixed with both liquid and solid food. Flies were starved for 2 h prior to exposure, then orally administered mock or SARS-CoV-2 solutions supplemented with 5% (w/v) BPB. Groups of 10 flies were housed per vial. To facilitate accurate quantification and avoid underestimation due to superimposed spots, flies were transferred to fresh vials at 1 and 4 hpi. Fecal output was quantified by counting the number of blue excreta spots deposited on the vial walls at 1, 4, and 12 hpi. Counts from each vial were treated as independent data points, and experiments were performed with eight biological replicates.

#### Lipid droplet staining

Guts dissected for lipid droplet staining were first fixed in 4% paraformaldehyde in PBS for 30 minutes, followed by 3 rinses in PBS. They were then permeabilized with 0.1% PBS-Triton X-100 for 5 minutes. For staining, the guts were incubated with a freshly prepared 1 μM solution of Nile Red (Sigma, Cat#: 72485) in PBST for 5 minutes or with an Oil Red O (Sigma,O0625-25g) working solution for 20 minutes. The Oil Red O working solution was prepared fresh as a 6:4 dilution of a 0.1% stock solution in isopropanol with distilled water, then filtered through a 0.45 μm syringe filter. After staining, the guts were washed twice and mounted in a PBS: Glycerol (1:1) mixture. Midguts and A549-ACE2 cells were stained with Bodipy FL C12 according to manufacturer’s instructions (Invitrogen, L3483). Imaging for Nile Red and Bodipy FL C12 staining was performed using a Nikon C2si confocal microscope with NIS Elements software. Mean fluorescence intensity of both dyes was quantified using ImageJ 1.52a, from image stacks containing the same number of slices, and normalized to DAPI signal.

#### Midgut pH assay

To assess changes in gut acidity following SARS-CoV-2 exposure, flies were fed a solution containing 0.1% (w/v) Phenol Red (Sigma), mixed with either mock or viral suspensions. Flies were starved prior to feeding to enhance ingestion efficiency. Successful dye ingestion was confirmed by the visible presence of red coloration in the abdomen within minutes of exposure. Only flies with clear abdominal dye accumulation were selected for subsequent analysis. At 4 hpi, flies were dissected, and midguts were immediately imaged using a Nikon SMZ18 stereomicroscope. As gut pH is highly sensitive to dissection and rapidly changes *ex vivo*, all imaging was performed within minutes to preserve physiological pH profiles.

#### Gut RNA-seq analysis

Following the virus ingestion steps, 12 guts per replicate including the crop, midgut, hindgut, and rectum were dissected in sterile RNAse free PBS. The study examined multiple timepoints: 4, 12, 24, and 48 hpi, with three independent replicates for each condition.

Like viral exposure protocol, bacterial oral infection was carried out at 29°C by using vials in which the fly food was covered by a Whatman filter paper containing 150 μl of either 2.5% sucrose solution (control), or mixed solution of 75 μl 5% sucrose with equal volume OD600 = 200 bacterial pellets. Midguts were dissected in PBS 1X at 4, 12, and 48 hpi.

Samples were homogenized in RNAse-free tubes with 500 μL of TRIzol and subsequently stored at -80°C. Next, total RNA was extracted via a modified phenol-chloroform method.^54^ Briefly, samples were thawed on ice and 120 μL of chloroform were added. Samples were vortexed and centrifuged for 15 min at 12,000X g, at 4°C. Next, the aqueous supernatant phase was transferred to a fresh tube, where it was mixed with 700 μL Buffer RLT (Qiagen RNeasy kit, cat #74004), and then with 500 μL of 100% ethanol. 700 μL at a time of the mixture were passed through a RNeasy spin column and centrifuged for 20 s at 10,000X g, followed by a final spin for 1 minute at 10,000X g to dry the column. The membrane was then washed twice with 500 μl of RPE and centrifuged for 20 s at 10,000X g, and centrifuged for 1 minute at 16,000X g to dry the column. Samples were eluted in 30 μl of RNase-free water.

Libraries were prepared using the Quantseq 3’ mRNA-seq prep kit from Lexogen according to the manufacturer’s instructions. Sample quality was evaluated before and after library preparation using a fragment analyzer (Advanced Analytical). Libraries were pooled and sequenced on the Illumina Nextseq 500 instrument using standard protocols for 150 bp single end read sequencing. Sequencing was carried out at Cornell University BRC Genomics Core Facility (RRID:SCR_021727). Raw sequence data have been deposited at Sequence Read Archive (SRA PRJNA1137395 for *Ecc15* and SRA PRJNA1137405 for SARS-CoV-2) and are publicly available. Reads were mapped to the host transcriptome (r6.55) using Salmon and differences in expression levels after ingestion were calculated using DESeq2 in R. Gene Ontology over-representation analysis was performed using g:Profiler.^55^ Kyoto Encyclopedia of Genes and Genomes (KEGG) pathway enrichment analysis was performed on the subset of significantly modulated genes involved in lipid metabolism related GOs using DAVID online ressources. PCA plots, Volcano plots, Venn diagrams, GO bubble plots, correlation plots, clustering (heatmap) of gene expression, and chordDiagrams were generated in R using ggplot2, DEseq2, pheatmap, and circlize packages. Transcriptomic profiles from 4 hpi and 12 hpi samples were compared to 4 hpi mock controls, while data from 24 hpi and 48 hpi samples were compared to 24 hpi mock controls.

#### Cytotoxicity assay

Cell viability following Plitidepsin treatment was assessed *in vitro* using ATP assay (CellTiter-Glo 2.0 Cell Viability Assay from Promega). This assay was performed on A549-ACE2 cells. Cells were cultured in 96-well plates at a density of 2×10^4^ cells per well in DMEM 10% FBS. The following day, ten-fold dilutions series of Plitidepsin ranging from 10 μM to 10^-6^ μM were used to treat cells. At 24- and 48-hours posttreatment, cells were rinsed with PBS, and 100 μL of culture medium mixed with 50 μl ATP reagent. After 2 min incubation time, cells were mechanically lysed, and ATP release was revealed by measuring luminescence.

#### Flow cytometry assay

Cells were rinsed twice with PBS 1X, detached with 20 μL of trypsin/EDTA, and then fixed with 3.7% paraformaldehyde in MEM 5% FBS solution for 15 minutes. After PBS wash, the cells were next incubated for 1 hour with the monoclonal human IgG1 anti-Spike antibody (Invivogen), diluted in 0.1% PBS-Triton X-100. Detection of the primary antibody was accomplished using a secondary goat anti-Human IgG antibody conjugated with Alexa Fluor 488 (Thermo Fisher Scientific), allowing the identification of SARS-CoV-2-infected cells. Following washing with PBS-Triton X-100, samples were subjected to flow cytometric analysis using Cytoflex (Beckman Coulter, Brea, CA, USA). Quantitative analyses were realized using CytExpert software.

### Quantification and statistical analysis

Data analysis and graph generation were performed using RStudio (R Statistical Software v4.2.0). At least, three biological replicates were performed for all experiments. Quantification data were first assessed for normality (Shapiro test) and homogeneity (Bartlett’s test). Results were then analyzed using one-way or two-way ANOVA followed by Tukey’s posthoc-tests, or Kruskal-Wallis followed by Dunn’s posthoc-test, Mann-Whitney U-test, or Student’s t-test (ns p>0.05, ∗p < 0.05, ∗∗p < 0.01, ∗∗∗p < 0.001, ∗∗∗∗p < 0.0001). Boxplots include 0.25, 0.5, 0.75 quantiles and mean represented by black lines. Line graphs and histograms were expressed as mean ± SD. The 50% cytotoxic (CC_50_) and inhibitory (IC_50_) concentrations were estimated using nonlinear regression to generate sigmoidal dose-response curves. For IC_50_, values are normalized against control groups of infected but untreated cells.

## Supplemental information

Document S1. Figures S1–S7

Table S1. List of significantly differentially expressed genes upon SARS-CoV-2 ingestion

Table S2. Correlation analysis of genes regulated following SARS-CoV-2 versus Ecc15 oral infection

Table S3. List of core upregulated and downregulated genes upon SARS-CoV-2 ingestion

Table S4. Gene Ontology enrichment analysis of genes modulated by SARS-CoV-2 in the midgut
