# Supplementary material for: Comprehensive investigation of SARS-CoV-2 intestinal pathogenesis in Drosophila
Source: iScience. 2026 Apr 27;29(5):115621. doi: 10.1016/j.isci.2026.115621 (PMC13138061; doi:10.1016/j.isci.2026.115621)
Supplement: Document S1. Figures S1–S7 [file mmc1.pdf]

iScience, Volume *29*

Supplemental information

Comprehensive investigation of SARS-CoV-2 intestinal pathogenesis in *Drosophila*

Layla El Kamali, Peter Nagy, Justine Girard, Nicolas Buchon, Patrick Mavingui, Chaker El-Kalamouni, and Dani Osman

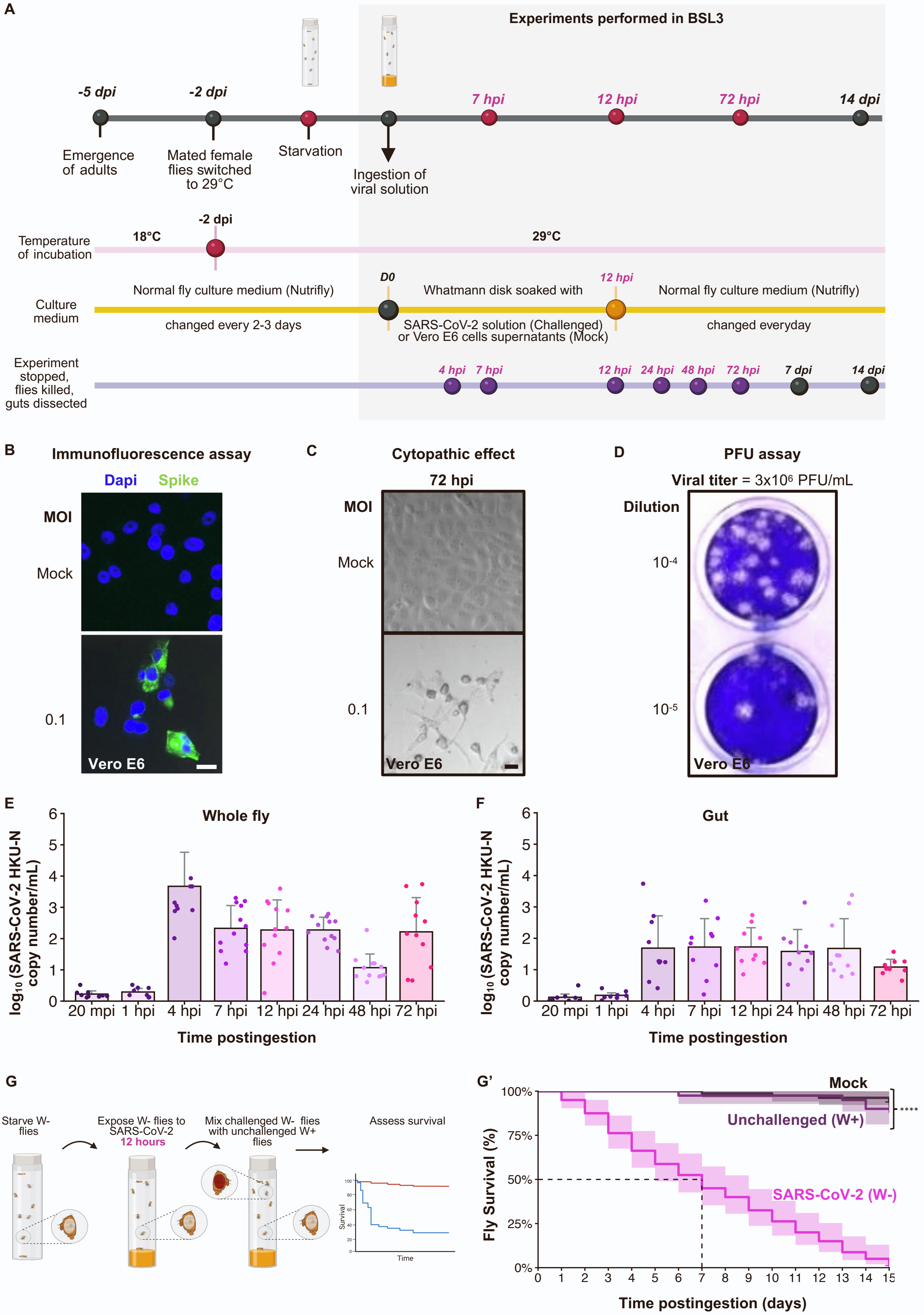
A
Experiments performed in BSL3
-5 dpi
-2 dpi
7 hpi
12 hpi
72 hpi
14 dpi
Emergence of adults
Mated female flies switched to 29°C
Starvation
Ingestion of viral solution
Temperature of incubation
-2 dpi
18°C
29°C
Culture medium
Normal fly culture medium (Nutrifly)
changed every 2-3 days
D0
Whatmann disk soaked with
SARS-CoV-2 solution (Challenged) or Vero E6 cells supernatants (Mock)
12 hpi
Normal fly culture medium (Nutrifly)
changed everyday
Experiment stopped, flies killed, guts dissected
4 hpi
7 hpi
12 hpi
24 hpi
48 hpi
72 hpi
7 dpi
14 dpi
B
Immunofluorescence assay
Dapi
Spike
MOI
Mock
0.1
Vero E6
C
Cytopathic effect
72 hpi
MOI
Mock
0.1
Vero E6
D
PFU assay
Viral titer = 3x10^6 PFU/mL
Dilution
10^-4
10^-5
Vero E6
E
Whole fly
log10 (SARS-CoV-2 HKU-N copy number/mL)
0
1
2
3
4
5
6
20 mpi
1 hpi
4 hpi
7 hpi
12 hpi
24 hpi
48 hpi
72 hpi
Time postingestion
F
Gut
log10 (SARS-CoV-2 HKU-N copy number/mL)
0
1
2
3
4
5
6
20 mpi
1 hpi
4 hpi
7 hpi
12 hpi
24 hpi
48 hpi
72 hpi
Time postingestion
G
Starve W- flies
Expose W- flies to SARS-CoV-2
12 hours
Mix challenged W- flies with unchallenged W+ flies
Assess survival
Survival
Time
G'
Mock
Unchallenged (W+)
SARS-CoV-2 (W-)
****
Fly Survival (%)
100%
75%
50%
25%
0%
0
1
2
3
4
5
6
7
8
9
10
11
12
13
14
15
Time postingestion (days)

Figure S1. *Drosophila* oral infection approach with an infectious SARS-CoV-2 clinical isolate

(A) Diagram summarizing the global approach to SARS-CoV-2 enteric exposure in *Drosophila*. Ingestion occurs on day 0 (D0), with flies feeding on viral solution for 12 hpi before being transferred to normal medium. Fly preparation starts 5 days prior to ingestion (-5 dpi), and some experiments last till 14 dpi. Created with BioRender.

(B) Immunohistochemical staining of Vero E6 cell monolayers with anti-SARS-CoV-2 spike antibody (green) and DAPI (blue). Cells were observed under 10x magnification using fluorescent microscopy at 48 hpi. The top row shows mock or uninfected cells, while the bottom row shows cells infected with a multiplicity of infection (MOI) of 0.1. Scale bar represents 10 µm.

(C) Cytopathic effect observed in Vero E6 cells infected with a SARS-CoV-2 clinical isolate. Cell cultures were observed under 10x magnification using light microscopy at 72 hpi. The top row shows uninfected cells, while the bottom row shows cells infected with SARS-CoV-2 at an MOI of 0.1. Scale bar represents 10 µm.

(D) Quantification of SARS-CoV-2 viral production by PFU assay. Images display plaque morphology, size, and halo structure, characteristic of SARS-CoV-2. Two representative images are presented: the top row shows the 10^{-4} dilution, and the bottom row shows 10^{-5} dilution of the viral stock.

(E) Longitudinal detection of SARS-CoV-2 genomic RNA using the HKU Nucleocapsid (HKUN) RT-qPCR assay. Samples consisted of pools of 5 ground whole flies or guts **(F)**. Three independent experiments were conducted with 4 samples each (n=12 samples/condition/timepoint). Histograms show means with error bars indicating standard deviation. Statistical analysis using one-way ANOVA revealed no significant differences in viral RNA levels over time.

(G) Schematic illustration summarizing the fly-to-fly transmission assay experimental approach. *W-* (white-eyed) flies were orally exposed to SARS-CoV-2 or mock solution for 12 h, following a 2 hour-starvation period, then co-housed with unchallenged *W+* (red-eyed) flies. Survival was subsequently monitored over time. Created with BioRender.

(G') Kaplan-Meier survival curves comparing *W-* (white-eyed) challenged flies that ingested the SARS-CoV-2 solution, *W+* (red-eyed) unchallenged flies co-housed with challenged ones, and *W-* flies that ingested Vero E6 cell supernatants. Data represent four independent replicates, with 20 *W-* and 10 *W+* flies per condition per replicate (total n= 80 *W-* or *40 W+* flies per condition). Shaded areas represent the 95% confidence intervals (CI). Statistical significance was measured using a global Log-rank test ($p<0.01$).

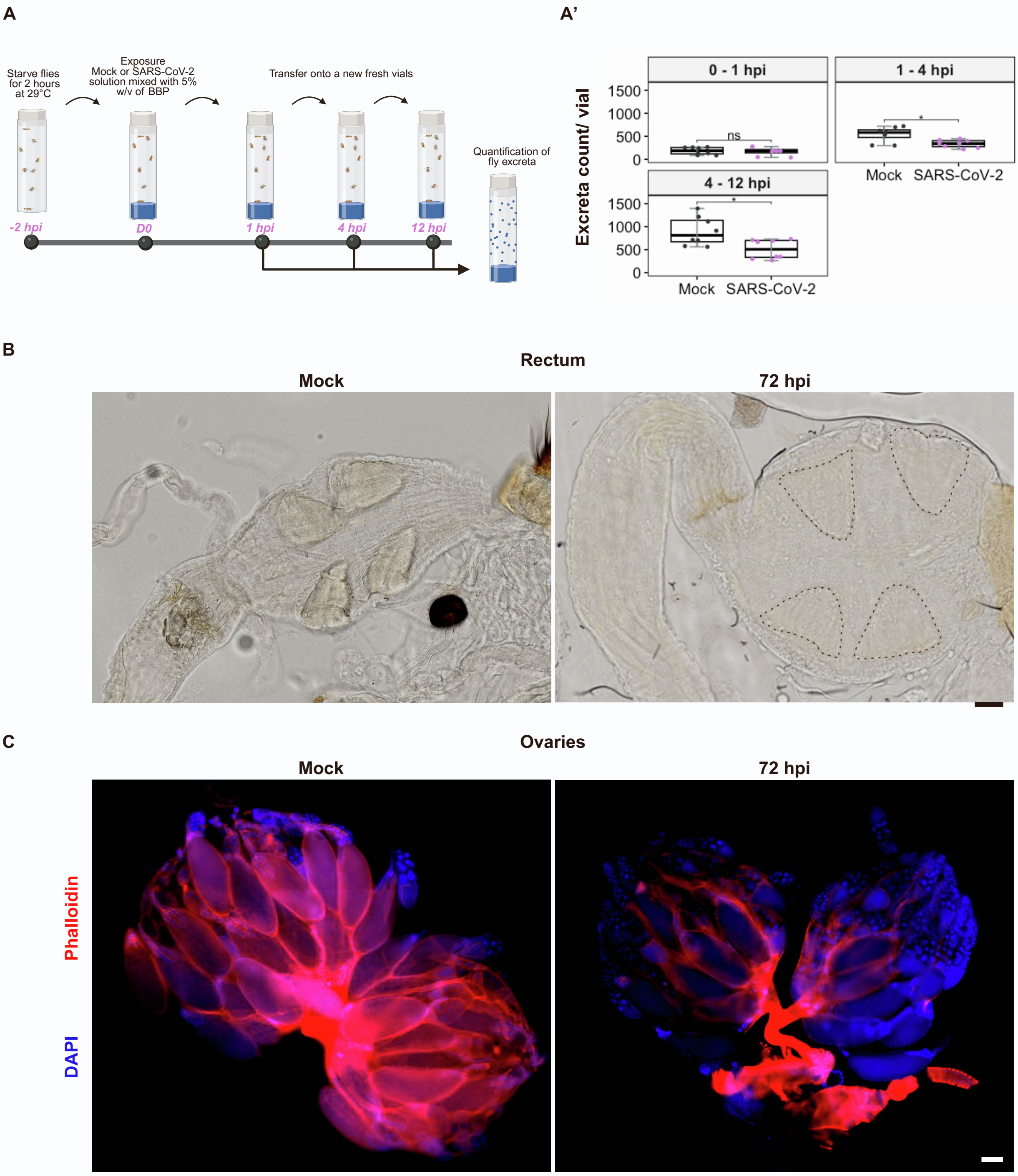
A
Starve flies for 2 hours at 29°C
Exposure Mock or SARS-CoV-2 solution mixed with 5% w/v of BBP
Transfer onto a new fresh vials
Quantification of fly excreta
-2 hpi
D0
1 hpi
4 hpi
12 hpi
A'
0 - 1 hpi
1 - 4 hpi
4 - 12 hpi
Excreta count/ vial
1500
1000
500
0
ns
*
Mock
SARS-CoV-2
B
Rectum
Mock
72 hpi
C
Ovaries
Mock
72 hpi
Phalloidin
DAPI

Figure S2. SARS-CoV-2 induces structural disruptions in multiple *Drosophila* organs

(A) Schematic diagram of the excretion quantification assay. Flies were exposed to mock or SARS-CoV-2 solution supplemented with bromophenol blue (BPB) at a concentration of 5% w/v. To ensure accurate quantification, flies were transferred to fresh vials every other hour. Fecal output was monitored by counting blue excreta spots on the vial edges at defined time points. Created with BioRender. **(A')** Quantification of the number of blue excreta spots. Data were collected from eight independent replicates with 10 flies per vial. Each dot represents the excreta count from one vial. P-values from simple t-tests corrected upon Bonferroni method are ns>0.05, and * <0.05.

(B) Rectums of infected flies observed under a stereoscope showed structural aberrations at 72 hpi compared to control. The dotted lines in the infected rectum delimit the rectal papillae. Scale bar represents 20 µm.

(C) Ovaries of infected flies observed under a Nanozoomer microscope at 72 hpi showed a disorganized and atrophic structure compared to control. Stained with DAPI (blue) and phalloidin (red). Scale bar represents 100 µm.

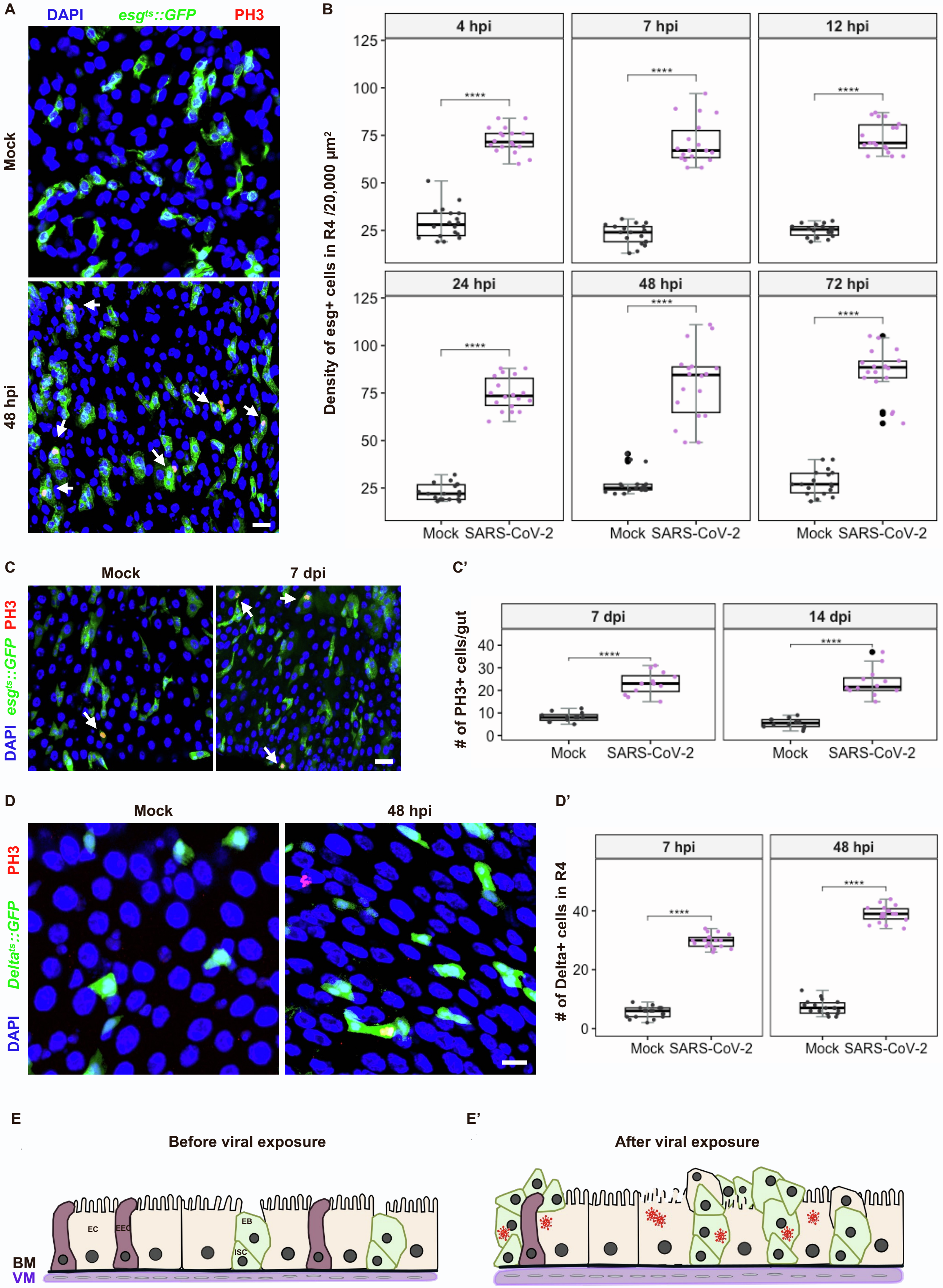

A
DAPI
esgts::GFP
PH3
Mock
48 hpi
B
4 hpi
7 hpi
12 hpi
24 hpi
48 hpi
72 hpi
Density of esg+ cells in R4 /20,000 µm²
125
100
75
50
25
****
Mock SARS-CoV-2
C
Mock
7 dpi
DAPI esgts::GFP PH3
C'
7 dpi
14 dpi
# of PH3+ cells/gut
40
30
20
10
0
Mock SARS-CoV-2
D
Mock
48 hpi
PH3
Deltats::GFP
DAPI
D'
7 hpi
48 hpi
# of Delta+ cells in R4
40
20
0
Mock SARS-CoV-2
E
Before viral exposure
EC
EEC
EB
ISC
BM
VM
E'
After viral exposure

Figure S3. Expansion of intestinal stem cells after SARS-CoV-2 enteric exposure in *Drosophila*

(A) Representative images of the R4 midgut region at 48 hpi, showing ISCs and EBs (*esg^{ts}::GFP*, green), and mitotic figures (PH3, red). Arrows point to PH3-positive cells.

(B) Density of esg-positive cells in a 20,000 μm^2 surface area of the R4 midgut region of SARS-CoV-2 infected flies and their controls at different times postingestion.

(C) Representative images of the R4 midgut region at 7 days postingestion (dpi), showing ISCs and EBs (*esg^{ts}::GFP*, green), and mitotic figures (PH3, red). Arrows point to PH3-positive cells. (**C'**) Quantification of mitotic ISCs (PH3-positive cells) per midgut at 7 and 14 days postingestion in surviving flies.

(D) Representative images of the R4 midgut region at 48 hpi and its respective mock, showing ISCs (*Delta^{ts}::GFP*, green) and mitotic figures (PH3, red). White arrows point to PH3-positive cells. (**D'**) Quantification of *Delta*-positive cells in the R4 region of SARS-CoV-2 infected flies and their respective controls at 7 and 48 hpi.

(E) Schematic diagram representing the structure of the intestinal epithelial monolayer and the major morphological changes observed after SARS-CoV-2 ingestion (**E'**). The basal membrane (BM) is represented in black, and the visceral muscle (VM) in purple. Dying cells are represented by dotted of segmented lines.

Data were collected from three independent replicates with 6 guts each (Total n=18 midguts/condition/timepoint). Each dot represents count from one gut. Large black dots mark outliers. P-values from simple t-tests corrected upon Bonferroni method (B, C') are **** <0.0001.

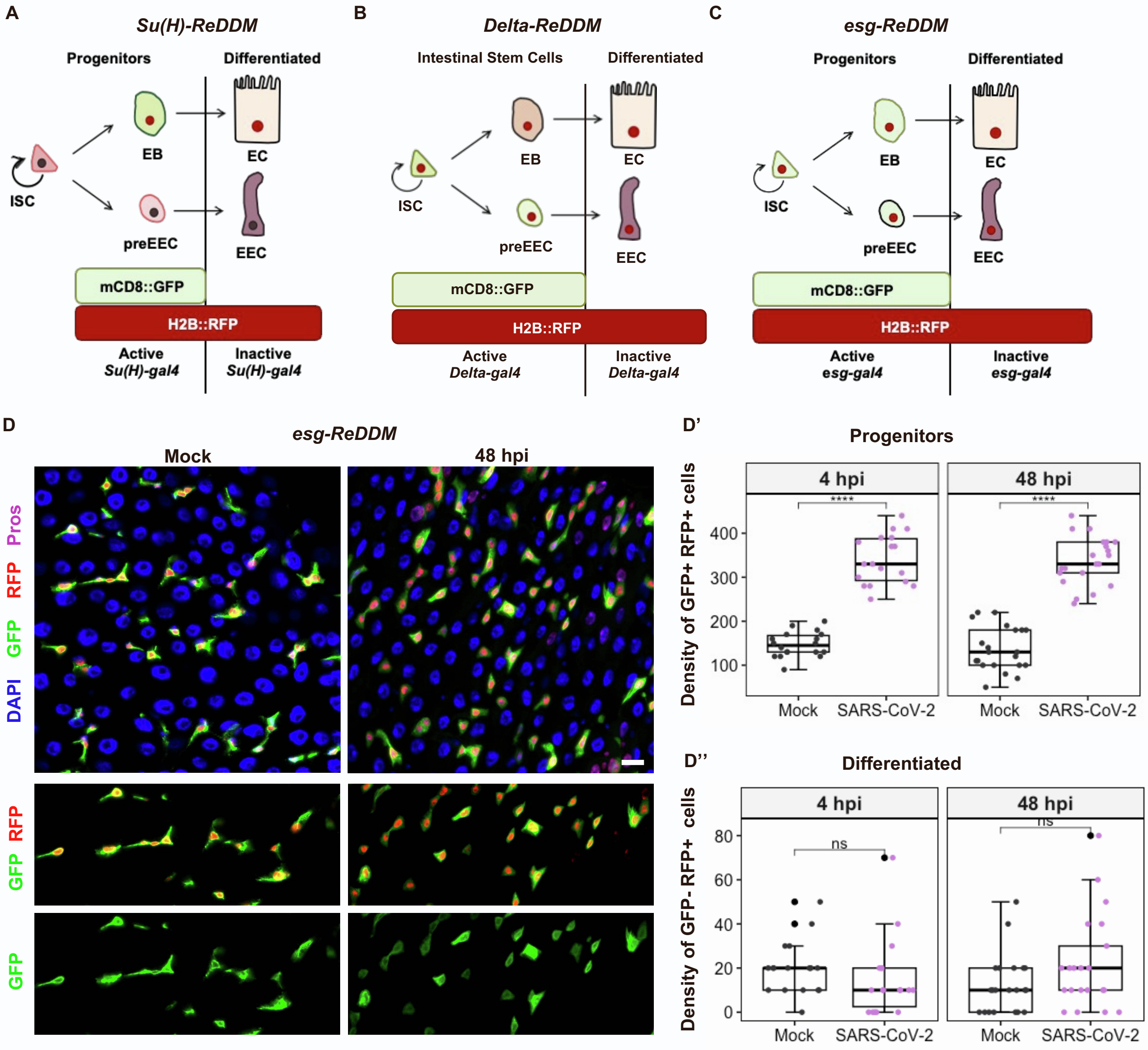

Figure S4. Lineage tracing systems for monitoring intestinal cellular turnover following SARS-CoV-2 ingestion

Schematic diagram of the intestinal lineage tracing systems used in this study: *(A) Su(H)-ReDDM*, (B) *Delta-ReDDM*, (C) *esg-ReDDM*. The ReDDM (repressible dual differential stability markers) relies on the differential stabilities of a pair of fluorescent proteins: the short-lived membrane tethered mCD8-GFP (green), and the long-lived histone tethered H2B::RFP (red).

(D) Representative images of *esg-ReDDM* at 48 hpi. Quantification of progenitors (D') (ISCs + EBs + preEEs = esg+ = GFP+ RFP+ cells) and (D") differentiated cells (EBs + ECs + EEs = RFP+ only cells). DAPI is blue, and the scale bar represents 20 μm. Images were acquired at 48 hpi under 40x magnification using confocal microscopy. Quantification was done in R4 at 4- and 48 hpi. Data were collected from three independent replicates with 6 midguts each (n=18 midguts/condition/timepoint). Each dot represents count from one midgut; large black dots mark outliers. P-values from the Mann Whitney U-test are ns>0.05, * <0.05, ** <0.01, *** <0.001, **** <0.0001.

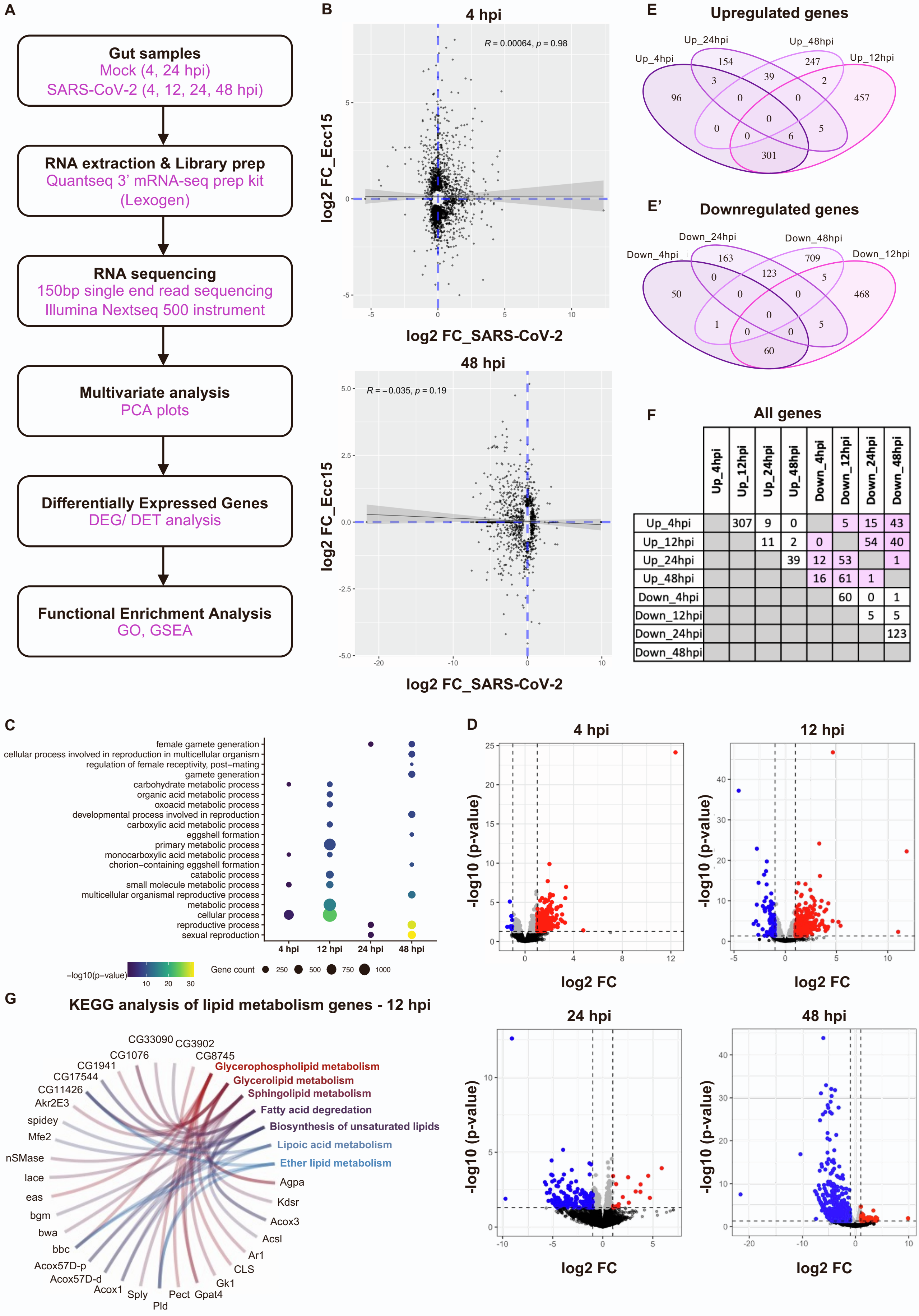
A
Gut samples
Mock (4, 24 hpi)
SARS-CoV-2 (4, 12, 24, 48 hpi)
RNA extraction & Library prep
Quantseq 3' mRNA-seq prep kit
(Lexogen)
RNA sequencing
150bp single end read sequencing
Illumina Nextseq 500 instrument
Multivariate analysis
PCA plots
Differentially Expressed Genes
DEG/ DET analysis
Functional Enrichment Analysis
GO, GSEA
B
4 hpi
R = 0.00064, p = 0.98
log2 FC_Ecc15
log2 FC_SARS-CoV-2
48 hpi
R = −0.035, p = 0.19
log2 FC_Ecc15
log2 FC_SARS-CoV-2
E
Upregulated genes
Up_4hpi
Up_24hpi
Up_48hpi
Up_12hpi
154
247
3
39
2
96
0
0
457
0
0
0
6
5
301
E'
Downregulated genes
Down_4hpi
Down_24hpi
Down_48hpi
Down_12hpi
163
709
0
123
5
50
0
0
468
0
1
0
0
5
60
F
All genes
Up_4hpi | Up_12hpi | Up_24hpi | Up_48hpi | Down_4hpi | Down_12hpi | Down_24hpi | Down_48hpi
Up_4hpi | | 307 | 9 | 0 | | 5 | 15 | 43
Up_12hpi | | | 11 | 2 | 0 | | 54 | 40
Up_24hpi | | | | 39 | 12 | 53 | | 1
Up_48hpi | | | | | 16 | 61 | 1 |
Down_4hpi | | | | | | 60 | 0 | 1
Down_12hpi | | | | | | | 5 | 5
Down_24hpi | | | | | | | | 123
Down_48hpi | | | | | | | |
C
female gamete generation
cellular process involved in reproduction in multicellular organism
regulation of female receptivity, post−mating
gamete generation
carbohydrate metabolic process
organic acid metabolic process
oxoacid metabolic process
developmental process involved in reproduction
carboxylic acid metabolic process
eggshell formation
primary metabolic process
monocarboxylic acid metabolic process
chorion−containing eggshell formation
catabolic process
small molecule metabolic process
multicellular organismal reproductive process
metabolic process
cellular process
reproductive process
sexual reproduction
4 hpi
12 hpi
24 hpi
48 hpi
−log10(p−value)
10 20 30
Gene count
250 500 750 1000
D
4 hpi
12 hpi
24 hpi
48 hpi
-log10 (p-value)
log2 FC
G
KEGG analysis of lipid metabolism genes - 12 hpi
Glycerophospholipid metabolism
Glycerolipid metabolism
Sphingolipid metabolism
Fatty acid degredation
Biosynthesis of unsaturated lipids
Lipoic acid metabolism
Ether lipid metabolism
CG33090
CG3902
CG1076
CG8745
CG1941
CG17544
CG11426
Akr2E3
spidey
Mfe2
nSMase
lace
eas
bgm
bwa
bbc
Acox57D-p
Acox57D-d
Acox1
Sply
Pld
Pect
Gpat4
Gk1
CLS
Ar1
Acsl
Acox3
Kdsr
Agpa

Figure S5. SARS-CoV-2 induces dynamic changes in *Drosophila* midgut transcriptome

(A) Schematic diagram of the experimental workflow of the comparative transcriptome analysis.

(B) Scatterplot comparing the log2 fold change of differentially expressed genes (DEGs) between *Erwina carotovora carotovora* (*Ecc15* - y axis) and SARS-CoV-2 (x axis) at 4 and 48 hpi.

(C) Gene ontology (GO) enrichment bubble plot of significantly regulated genes grouped by time postingestion. The plot shows the top 20 significantly enriched biological processes at 4, 12, 24, and 48 hpi, compared to mock conditions. Bubble size corresponds to the number of genes per category, and color intensity reflects statistical significance (–$\log_{10}$ p-value).

(D) Volcano scatter plot of differentially expressed genes at different times postingestion. Black dots represent genes where -1<log2 FC<1, red dots are significantly overexpressed genes (log FC>1, P-value<0.05), blue dots are significantly downregulated genes (log FC<-1, P-value<0.05), and gray dots are genes that are non-significantly different (P-value>0.05).

(E) Venn diagrams depicting the number of upregulated or (E') downregulated genes showing significant differential regulation (p-value<0.05) at different times postingestion.

(F) Table showing the number of genes in the intersection that are significantly differentially expressed at different times postingestion. Pink-shaded cells highlight consistently changing genes.

(G) Chord diagram showing the association between differentially expressed genes and lipid metabolic pathways during early SARS-CoV-2 enteric exposure. The diagram illustrates the connections between individual genes (left) and their associated metabolic pathways (right). Colors represent distinct lipid-related processes.

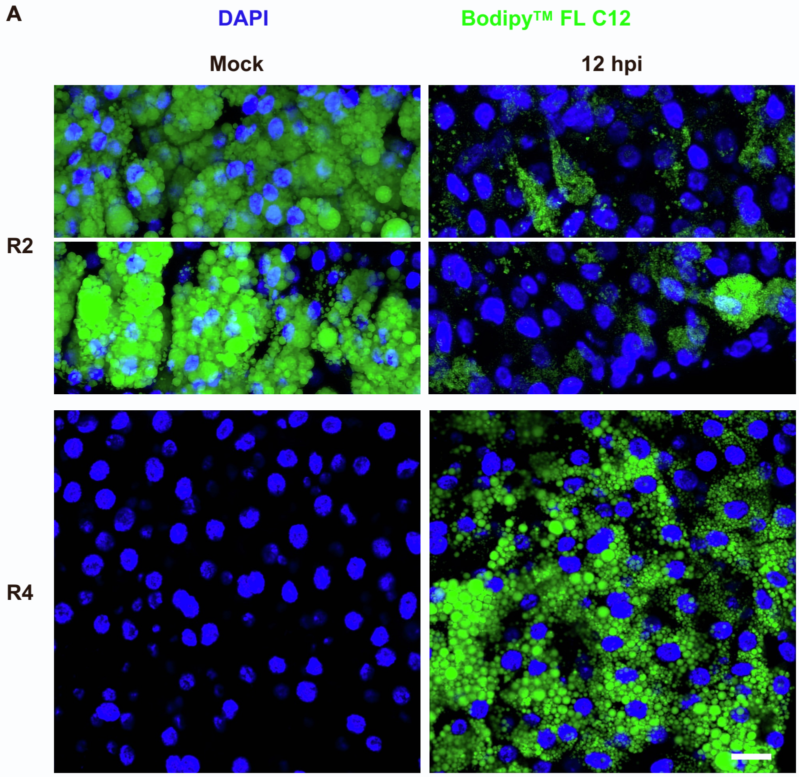

Figure S6. SARS-CoV-2 modulates lipid droplet distribution in *Drosophila* midgut

(A) Representative confocal images of w^{1118} *Drosophila* midguts stained with BODIPY™ FL C12 dye at 12 hpi compared to mock. Scale bar represents 20 µm.

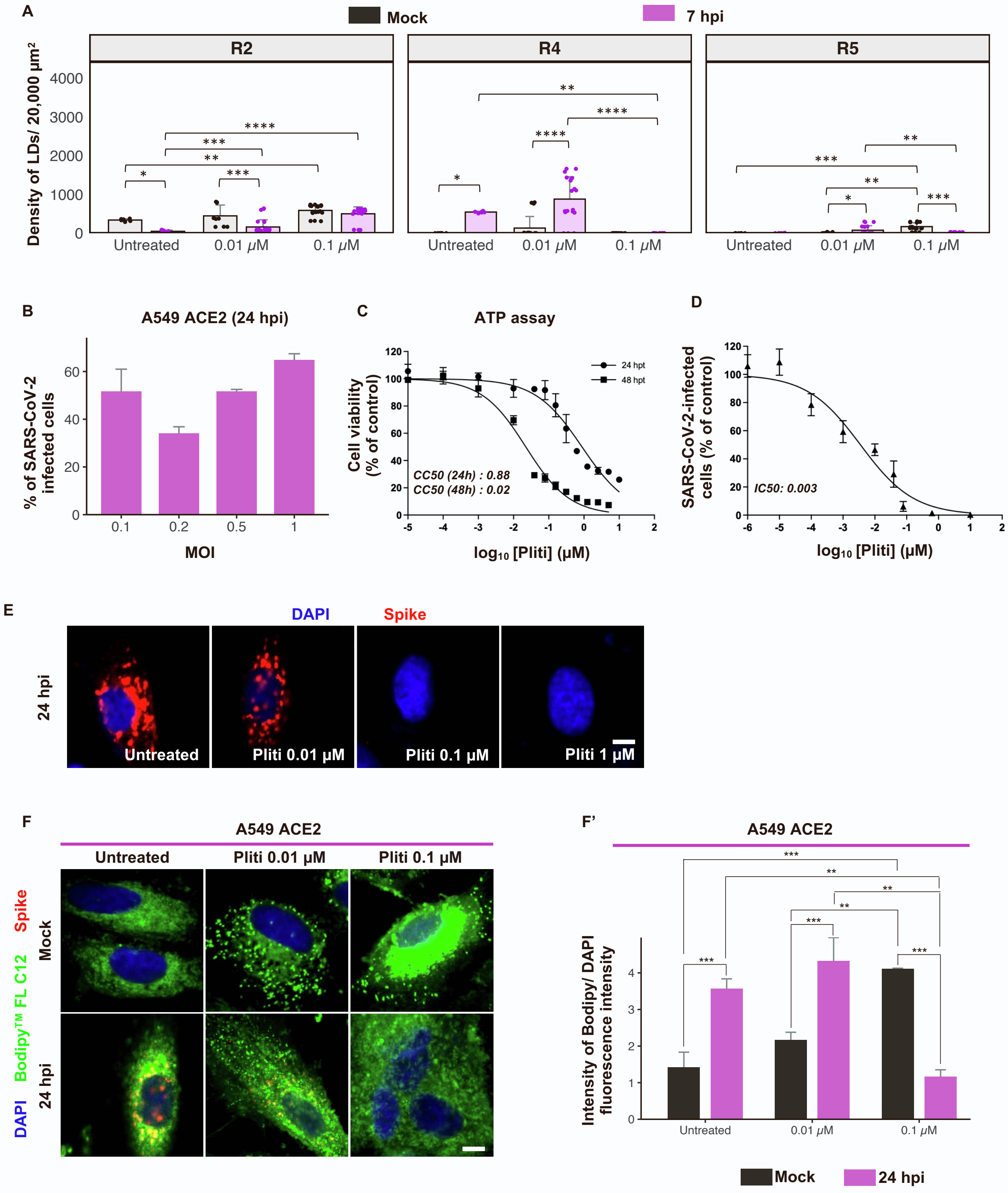
A
Mock
7 hpi
R2
R4
R5
Density of LDs/ 20,000 µm²
4000
3000
2000
1000
0
Untreated
0.01 µM
0.1 µM
B
A549 ACE2 (24 hpi)
% of SARS-CoV-2 infected cells
60
40
20
0
0.1
0.2
0.5
1
MOI
C
ATP assay
Cell viability (% of control)
24 hpt
48 hpt
CC50 (24h) : 0.88
CC50 (48h) : 0.02
log10 [Pliti] (µM)
D
SARS-CoV-2-infected cells (% of control)
IC50: 0.003
log10 [Pliti] (µM)
E
DAPI
Spike
24 hpi
Untreated
Pliti 0.01 µM
Pliti 0.1 µM
Pliti 1 µM
F
A549 ACE2
Untreated
Pliti 0.01 µM
Pliti 0.1 µM
Mock
24 hpi
DAPI Bodipy™ FL C12 Spike
F'
A549 ACE2
Intensity of Bodipy/ DAPI fluorescence intensity
4
3
2
1
0
Untreated
0.01 µM
0.1 µM
Mock
24 hpi

Figure S7. Plitidepsin counteracts SARS-CoV-2 activity in *Drosophila* midgut and human A549-ACE2 pulmonary cells

(A) Density of lipid droplet particles in a 20,000 μm^2 surface area of the R2-R5 midgut regions (related to Figure 7E-E") at 7hpi following cotreatment with 0.01 and 0.1 μM Plitidepsin, compared to controls (n=6 per condition per region). Histograms show means and error bars indicate standard deviation. P-values were defined using two-way ANOVA multiple comparisons (* P<0.05, ** P<0.01, *** P<0.001, and **** P<0.0001). Non-significant differences are not shown on the graph.

(B) Quantification of A549-ACE2 infected cells (%) at 24 hpi revealed by immunostaining using an anti-spike antibody at different multiplicities of infection (MOI).

(C) Cellular toxicity following Plitidepsin exposure at different concentrations, assessed via ATP assay at 24- and 48-hours posttreatment. The corresponding 50% cytotoxic concentrations (CC_{50}) are indicated. Data are expressed as mean of mean ± Standard deviation of the mean, normalized to control.

(D) Percentage of SARS-CoV-2 infected cells (MOI= 0,1) at 24 hpi following cotreatments with Plitidepsin at different concentrations, measured by flow cytometry assay using an anti-SARS-CoV-2-spike antibody. The 50% inhibition concentration (IC_{50}) is indicated. Results are represented as mean of mean ± standard deviation of the mean, normalized to control.

(E) Representative images of A549-ACE2 infected cells (MOI= 0,1) at 24 hpi, cotreated with 0, 0.01, 0.1, and 1 μM of Plitidepsin. Cells were stained with anti-spike antibody (red) and DAPI (blue).

(F) Representative images of lipid droplets in A549-ACE2 infected cells (MOI= 0,1) at 24 hpi compared to mock. Cells were cotreated with 0, 0.01, and 0.1 μM of Plitidepsin. Lipid droplets were stained with Bodipy™ FLC12 (green). Scale bar represents 30 μm. **(F')** Fluorescence intensity of Bodipy™ FLC12 quantified and normalized to that of DAPI. P-values were defined using two-way ANOVA (* P<0.05, ** P<0.01, *** P<0.001, and **** P<0.0001).
